# Mouse Hepatitis Virus Infection Upregulates Genes Involved in Innate Immune Responses

**DOI:** 10.1371/journal.pone.0111351

**Published:** 2014-10-31

**Authors:** Dhriti Chatterjee, Sankar Addya, Reas S. Khan, Lawrence C. Kenyon, Alexander Choe, Randall J. Cohrs, Kenneth S. Shindler, Jayasri Das Sarma

**Affiliations:** 1 Department of Biological Sciences, Indian Institute of Science Education and Research-Kolkata (IISER-K), Mohanpur, West Bengal, India; 2 Kimmel Cancer Centre, Thomas Jefferson University, Philadelphia, Pennsylvania, United States of America; 3 Scheie Eye Institute and FM Kirby Centre for Molecular Ophthalmology, University of Pennsylvania, Philadelphia, Pennsylvania, United States of America; 4 Departments of Anatomy, Pathology and Cell Biology, Thomas Jefferson University, Philadelphia, Pennsylvania, United States of America; 5 Departments of Neurology and Microbiology, University of Colorado School of Medicine, Aurora, Colorado, United States of America; Temple University School of Medicine, United States of America

## Abstract

Neurotropic recombinant strain of Mouse Hepatitis Virus, RSA59, induces meningo-encephalitis, myelitis and demyelination following intracranial inoculation. RSA59 induced neuropathology is partially caused by activation of CNS resident microglia, as demonstrated by changes in cellular morphology and increased expression of a microglia/macrophage specific calcium ion binding factor, Iba1. Affymetrix Microarray analysis for mRNA expression data reveals expression of inflammatory mediators that are known to be released by activated microglia. Microglia-specific cell surface molecules, including CD11b, CD74, CD52 and CD68, are significantly upregulated in contrast to CD4, CD8 and CD19. Protein analysis of spinal cord extracts taken from mice 6 days post-inoculation, the time of peak inflammation, reveals robust expression of IFN-γ, IL-12 and mKC. Data suggest that activated microglia and inflammatory mediators contribute to a local CNS microenvironment that regulates viral replication and IFN-γ production during the acute phase of infection, which in turn can cause phagolysosome maturation and phagocytosis of the myelin sheath, leading to demyelination.

## Introduction

There is a fundamental distinction between acute and chronic inflammation in various pathological studies. Acute inflammation comprises the immediate and early response to an injurious agent and is basically a defensive response that paves the way for repair of the damaged site. The term “neuroinflammation” is appropriate where limited neuronal insults trigger glial cell activation [1]. Neuroinflammation revolves around direct neuro-glial cell responses (host cell factors) which are induced by infection and injury within the CNS. It also involves the mechanism by which these responses ultimately contribute to neuropathology and neurobiology of diseases.

Microglia are the resident macrophage-like population in the central nervous system (CNS). Microglia remain quiescent in the CNS, unable to perform effector and APC functions until activated by injury or infection, and have been suggested to represent the first line of defence for the CNS, which normally lacks professional APCs until they are recruited to the CNS by inflammatory stimuli. Previous studies demonstrated that microglia can be persistently infected by neurotropic strains of Mouse hepatitis virus (MHV) [2].

Neurotropic MHV infection in mice causes meningoencephalitis, myelitis, and demyelination associated with pronounced activation of microglia. This is evident from characteristic changes in microglial cellular morphology and presence of abundant phagocytotic microglia in demyelinating plaques. MHV-induced CNS injury during early stage of infection involves microglia mediated neuroinflammation. But the mechanism by which these inflammatory responses ultimately contribute to MHV-induced demyelination and axonal loss is not clear. To understand neuroinflammatory pathways, expression of host inflammatory genes in MHV-infected mouse spinal cord is evaluated in comparison to non-infected mouse spinal cord by high throughput affymetrix microarray analysis. Brain and liver tissues from infected mice were evaluated for routine histopathological outcomes. Simultaneously, a high throughput Meso Scale Discovery (MSD)) Multi-Array Assay system for Mouse cytokines was performed to determine the predominant neuroinflammatory pathways involved in microglia-mediated myelin destruction.

## Material and Methods

### Ethics Statement

Use of animals and all experimental procedures were reviewed and approved by *the Institutional Animal Care and Use Committee* at the University of Pennsylvania, Philadelphia. Animal protocols adhered to the guidelines of the United States National Institutes of Health Office of Laboratory Animal Welfare Guide for the Care and Use of Laboratory Animals, 8^th^ Edition.

### Virus

RSA59 is recombinant isogenic demyelinating (DM) strain of MHV [3], [4]. RSA59 is isogenic to parental MHV-A59 but the spike gene is introduced by targeted RNA recombination. Spike gene encodes an envelope glycoprotein that mediates many biological properties of MHV including viral attachment to host cells, as well as virus-cell and cell-cell fusion [5]. These recombinant strains also express enhanced green fluorescence protein (EGFP) [3]. Briefly, the pMH54 plasmid (obtained from Paul S. Masters) comprises codon 28 of the hemagglutinin esterase (HE) pseudogene through to the 3′ end of the MHVA59 genome, including a poly(A) tail, as described by [6], used as transcription vector to generate RNA for targeted recombination as described previously [7], [8]. In order to replace gene 4 with the EGFP gene, pMH54 was modified by the introduction of a SalI site 42 nucleotides downstream of the intergenic sequence for gene 4a and a NotI site 102 bp upstream of the stop codon for gene 4b, using the Quick Change site-directed mutagenesis kit (Stratagene, La Jolla, CA). Targeted RNA recombination was carried out between synthetic capped RNAs transcribed from pMH54EGFP, using a T7 polymerase transcription kit (Ambion, Austin, TX) and fMHV as a recipient virus. Recombinant viruses in which the murine coronavirus spike gene has replaced the feline coronavirus spike gene were selected by replication in murine 17 Cl-1 cells [6], [9]. Candidate recombinants were plaque purified two times, and viral stocks were grown on 17Cl-1 cell for further characterization. For each desired recombinant, at least two viruses derived from independent recombination events were characterized. The EGFP containing viruses with the A59 spike were called RSA59 and used in this study.

### Inoculation of mice

Four-week-old, MHV-free, C57BL/6 (B6) mice (Jackson Laboratory) were inoculated intracranially with 50% LD_50_ dose of RSA59 strain (20,000 PFU) as described previously [10]. Five mice in each group were infected. Mice were monitored daily for signs of disease. Mock-infected controls were inoculated similarly but with an uninfected cell lysate at a comparable dilution. At day 6 post infection 3 mice were perfused with 40 ml of PBS 1x (treated with DEPC) and whole spinal cord, half of the brain and a piece of liver tissue were harvested for extraction of RNA and stored in RNA later solution at −80°C until extraction. The remaining liver and half brain were post fixed in 4% paraformaldehyde for parallel histopathological analysis. Two mice from each group were sacrificed for routine histopathological tissue processing. Two mice in control and three mice in the RSA59 infected group were analyzed in the microarray experiment. However, after the parallel histological analysis demonstrated that one of the RSA59 infected mice did not exhibit signs of infection, suggesting inoculation was unsuccessful, data from this mouse was removed from subsequent analyses.

### RNA extraction

RNA was extracted from 30 mg of brain, spinal cord and liver tissues from mock infected mice and viral infected mice with RNeasy mini kits (Qiagen, Chatsworth, CA) according to the manufacturer's instructions. The quantity of total RNA was assessed using a NanoDrop ND-100 spectrophotometer. One µg of total RNA was used to synthesize cDNA with high capacity cDNA archive kit (Applied Biosystems Inc., Foster, CA) according to the manufacturers' instructions for real time PCR and regular PCR analysis.

### Microarray experiment for mouse gene array

Total RNA was quantified on a NanoDrop spectrophotometer, followed by RNA quality assessment on an Agilent 2100 bioanalyzer (Agilent, Palo Alto, CA, USA). Amplification of cDNA was performed using the Ovation Pico WTA-system V2 RNA amplification system (NuGen Technologies, Inc.). Briefly, 50 ng of total RNA was reverse transcribed using a chimeric cDNA/mRNA primer, and a second complementary cDNA strand was synthesized. Purified cDNA was then amplified with ribo-SPIA enzyme and SPIA DNA/RNA primers (NuGEN Technologies, Inc.). Amplified ST-cDNA was purified with Qiagen MinElute reaction cleanup kit. The concentration of Purified ST-cDNA was measured using the Nanodrop. ST-cDNAs were fragmented and chemically labeled with biotin to generate biotinylated ST-cDNA using FL-Ovation cDNA biotin module V2 (NuGen Technologies, Inc.).

Briefly, Affymetrix gene chips, Mouse gene 1.0 ST array comprised of over 750,000 unique 25-mer oligonucleotide features constituting over 28000 gene-level probe sets (Affymetrix, Santa Clara, CA), were hybridized with fragmented and biotin-labeled target (2.5 µg) in 110 µl of hybridization cocktail. Target denaturation was performed at 99°C for 2 min and then 45°C for 5 min, followed by hybridization at 45°C for 18 h with 60 rpm in hybridization oven 645. Arrays were then washed and stained with the GeneChip hybridization, wash and stain kit using Gene chip Fluidic Station 450 according to Affymetrix standard protocol (www.affymetrix.com). Chips were scanned on an Affymetrix Gene Chip Scanner 3000 7G, using Command Console Software.

Background correction and normalization were done using Iterative Plier 16 with GeneSpring V12.0 software (Agilent). 1.5-fold and 2-fold (p-value <0.05) differentially expressed gene lists were generated. The list of differentially expressed genes was loaded into Ingenuity Pathway Analysis (IPA) 8.0 software (http://www.ingenuity.com) to perform biological network and functional analyses.

### PCR and Real Time PCR

Quantitative Real-Time (RT)-PCR was performed on the ABI PRISM 7000 Sequence Detection System using TaqMan Universal PCR Master Mix (Applied Biosystems) and TaqMan Gene Expression Assays primer/probe (Applied Biosystems; Assay IDs are listed in Table 1) according to the manufacturer's instructions. β- actin (ACTB) primer was used as internal control. Samples were analyzed in triplicate. Amplification data were analyzed with ABI prism sequence Detection Software 2.1 (Applied Biosystems). For regular PCR a pair of oligonucleotide primers IZJ (5′–GCTCCACAGTTGGTGCC-3′) and IZJ6 (5′-ACGTAGGACCTTGCTAACTTC-3′) was designated for PCR amplification (data not shown). The reaction consisted of 3 min of denaturation at 94°C, 1 min of denaturation at 94°C, 45 s of annealing at 55°C, and 2 min of extension at 65°C. After 30 cycles, the final products were extended for 5 min at 72°C. The resulting amplified fragment of 601 BP was analyzed by 1.2% agarose gel electrophoresis. Genes used for RT-PCR quantification are shown in Table 1.

**Table 1:** Table showing the list of primers used for Real Time PCR Analysis.

Gene Name	Sequence
CD4	Forward Primer – CTGACTCTGACTCTGGACAA
	Reverse Primer- TGAGCTGAGCCACTTTC
	Probe: AGTGAACCTGGTGGTGATG
CD8	Forward Primer – TACTTCTGCGCGACGGTT
	Reverse Primer – GCAGTTGTAGGAAGGACATC
	Probe – ACGAAGCTGACTGTGGTTGA
Aif1	Forward- GCCTGTCTTAACCTGCATCA
	Reverse- AGGCATCACTTCCACATCAG
	Probe - TGAGGAGATTTCAAAAGC
ITGAM (CD11b)	Forward- ACAGCATCAGTACCAGTTCA
	Reverse- GATCCCATATGGTCACATTG
	Probe- ATCCCTGTTCAGATCAAC
CD52	Forward- AAGCAGCCAGGTTCAAAGTG
	Reverse- ATCCTGTTTGTATCTGAATC
	Probe – CATTCCTTCTGGTTGTGA
CD74	Forward- TGCTGATGCGTCCAATGTC
	Reverse- TACTCCAGGGGTCCAGA
	Probe – CATCTGCTCACGAGGTC
CD68	Forward- TAGGACCGCTTATAGCCCAAG
	Sequence
	Reverse- CTGTAGGTGTCATCGTGAAG
	Probe – GTTACTCTCCTGCCATCC
CD19	Forward- ATTGTCTCCGAGGAAACCTG
	Reverse- AGTTCTCAACAGCCAGAG
	Probe – AAGGTCAGCAGTGTGGC
ACTB	Forward- CTTCTACAATGAGCTGCGTGTG
	Reverse- GGTCTCAAACATGATCTGG
	Probe – CCGTGAAAAGATGACCC
Irgm1	Forward Primer – AGACAGCGTCACTCGGAT
	Reverse Primer- AGTAGTGGAGCAGCCTCGCA
	Probe – ATCACACAGTTCCTGCG
Irgm2	Forward Primer – ACCGTCCTGGAGCCTGGATT
	Reverse Primer – TTGTCGAGCAACGGGGCAAC
	Probe- GGTTCTGAGCAGGTTG
Iigp1	Forward- GAAGCAGATGGCAAACCTC
	Reverse- TCCCTAAAGGTGTTCAC
	Probe – GGACATCCGCCTTAACTGTG
CXCL9	Forward- GAACTCAGCTCTGCCATGAAG
	Reverse- ACTAGGGTTCCTCGAACT
	Probe – CCTGGAGCAGTGTGGAG
	Sequence
CXCL10	Forward- CGTGTTGAGATCATTGCCACGA
	Reverse- GGAGCCCTTTTAGACCTTT
	Probe – TGAAAGCGTTTAGCCAAAA
GBP2	Forward- ACGTAGGACCTTGCTAACTTC
	Reverse- CGAGCTGATGAGACATCCAT
	Probe – TTATCAAGAAGAACATGG
GBP10	Forward- CTGTGCAGTCTCAAACCAAG
	Reverse- CACAAGTCGTTCTTAGG
	Probe- GATGTGGAAAAGGGTGATCC
Viral Primer	Forward- GCTCCACAGTTGGTGCC
	Reverse- ACGTAGGACCTTGCTAACTTC

### Histopathological Analysis

Mice were sacrificed at day 6 post-inoculation, and liver, brain and spinal cord tissues were harvested. Spinal cords were kept aside for RNA work while brain and liver tissues were used for histopathological studies. 5 µm thin sections were taken from liver and brain, stained with H& E for routine histological studies [11]. Serial sections were further immunostained with anti nucleocapsid antibody and anti Iba1 antibody (microglia/macrophage marker) as in prior studies [12].

### Protein Mesoscale Studies

Brain and spinal cord tissues were harvested from mice 6 days post-inoculation with RSA59. Mock infected mice were also sacrificed in parallel and brain and spinal cord tissues were harvested. The tissues were weighed and flash frozen in liquid nitrogen. During extraction, 150 mg of tissue was lysed in 1 ml of lysis buffer containing protease inhibitor cocktails. The sample was homogenized and centrifuged at 14000 rpm for 10 min at 4°C to remove debris. The protein concentration was determined and then the protein sample was loaded onto the Mesosclae Discovery Multiarray assay (MSD) plate.

MSD assays provide a rapid method for measuring the levels of multiple protein targets within a single volume sample. In a multiplex assay, an array of capture antibodies against different targets is patterned on distinct spots in the same well. The mouse Th1/Th2 9-Plex Assay detects IFN-γ, IL-1β, IL-2, IL-4, IL-5, KC/GRO, IL-10, IL-12 total and TNF-α in a sandwich immunoassay format. MSD provides a plate that has been pre-coated with capture antibodies on spatially distinct spots with an electro chemiluminiscent compound MSD SULFO TAG label. Analytes in the sample bind to the capture antibodies immobilized on the working electrode surface; recruitment of the detection antibodies by bound analytes completes the sandwich. MSD read buffer is added that provides the appropriate chemical environment for electrochemiluminescence. The plate is loaded into an MSD sector instrument for analysis. Inside the sector instrument, a voltage is applied to the plate electrode which causes the labels bound to the electrode surface to emit light. The instrument measures intensity of emitted light to afford a quantitative measure of the antibodies. Another single-plex plate containing antibody against IL-6 was also used to detect the presence and quantity of IL-6 in the protein sample.

### Data Analysis

Data analyses were performed GeneSpring software version 12.0 (Agilent Technologies, Inc., Santa Clara, CA). The probe set signals were calculated with the Iterative Plier 16 summarization algorithm; baseline to median of all samples was used as baseline option. Data was filtered by percentile and lower cut off was set at 25. The criteria for differentially expressed genes were set at ≥ 2-fold and 1.5-fold changes. Statistical analysis was performed to compare 2 groups using unpaired T Test with p-value less than or equal to 0.05. Heat maps were generated from differentially expressed gene list. The list of differentially expressed genes was loaded into Ingenuity Pathway Analysis (IPA) 8.0 software (http://www.ingenuity.com) to perform biological network and functional analyses.

## Results

### RSA59 induces acute neuropathology in mice

Four week old C57BL/6 mice were inoculated with RSA59 or mock-infected with PBS as controls. Three mice were sacrificed 6 days later, the time previously noted to result in peak CNS inflammation [13]. Sections of brain and liver, stained by H&E and immunostained for viral antigen and the microglial marker Iba1, confirmed successful inoculation in two of the RSA59 infected group by presence of encephalitis and hepatic inflammation in RSA59-infected mice, and no inflammatory lesions in control mice (Figure S1), similar to prior studies [14].

RNA was extracted from spinal cords of the same RSA59-infected and control mice. Relative expression levels of RNA transcripts were determined by quantitative (RT)-PCR using primer/probe designed specifically from MHV nucleocapsid, and β-actin primer was used as internal control. Samples were analyzed in triplicate. Amplification data (Figure S1 I), corroborates pathology data, showing large numbers of PCR transcripts present in two of the RSA59 infected mice, whereas no amplification was observed in control mice and very little amplification was observed in one of the infected mice.

### Innate Immunity Related Genes Are Upregulated due to RSA59 infection

**Figure 1:**
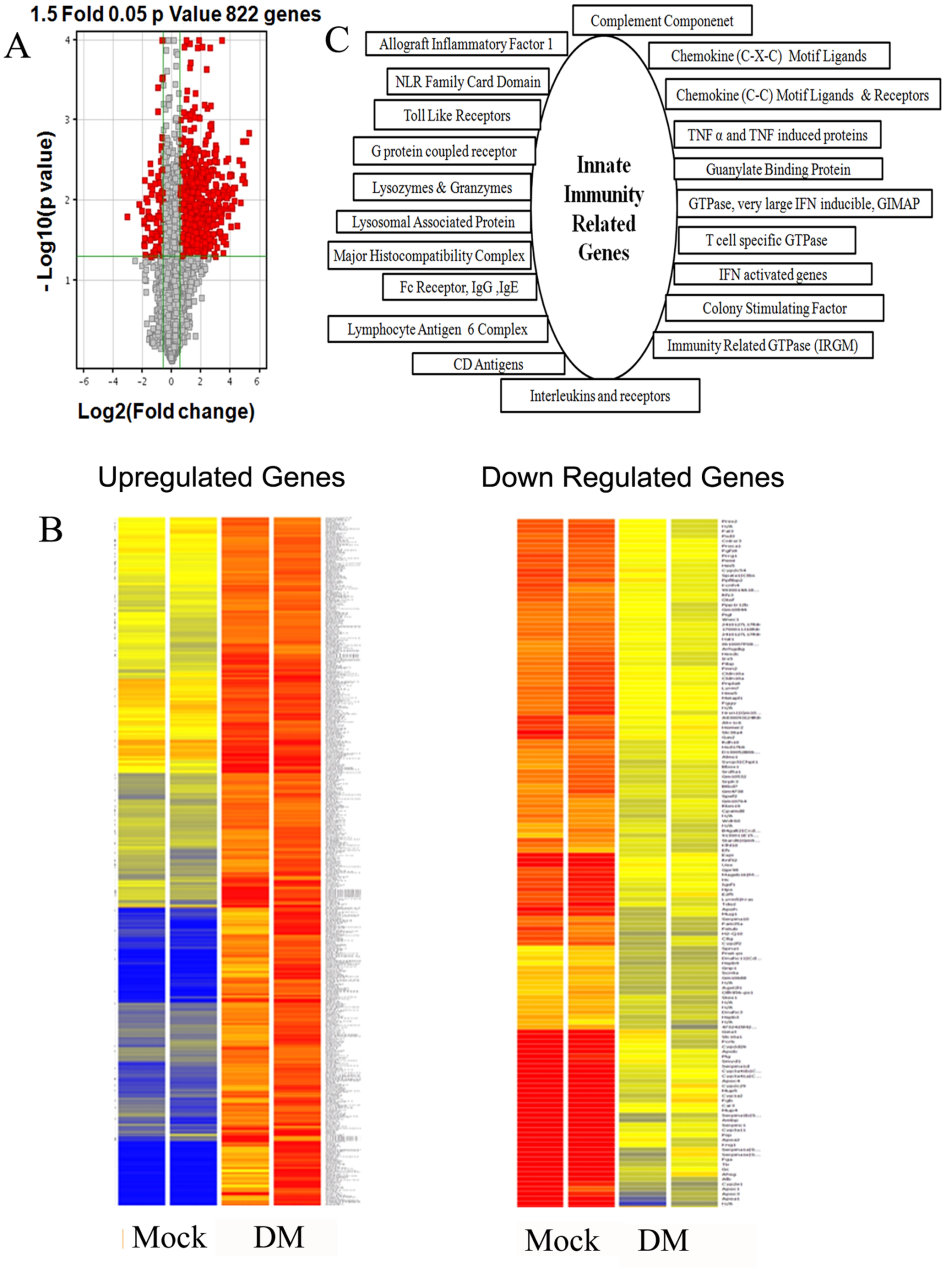
Volcano Plot of differentially expressed transcripts of two RSA59- (DM) infected mouse spinal cords at day 6 post-infection compared to two control mock-infected mouse (Mock) spinal cords (A). X-axis represents the log2 values of the fold change observed for each mRNA transcript while the Y-axis represents the log 10 values of the p-values of the significance tests between replicates for each transcript. Genes that are up- or down-regulated more than 1.5-fold in RSA59-infected mice compared to control mice are displayed in red. Heat Map of differentially up-regulated and down-regulated genes (B). Heat map of the 822 genes shown in red in A, shows 681 genes are up-regulated while only 141 genes are down-regulated in RSA59 infection (DM) in mice compared to mock-infected controls. The color code/dendrogram shows the expression level increasing from blue in control and gradually moving towards red in infected mice in cases of up-regulated genes, and vice versa in down-regulated genes (blue in infected mice and gradual shift to red in control mice). Functional cluster of genes involved in innate immune responses that were differentially expressed by more than 1.5-fold in RSA59-infected spinal cords compared to mock-infected spinal cords (C). Upregulated genes belong to several gene families known to be involved in apoptosis, microglial activation, complement signaling, soluble factors like cytokines and chemokines, colony stimulating factors, IFN-induced and activated genes, immunity related GTPases, guanylate binding proteins and major histocompatibility complex activation. Detailed differential expression levels (fold changes) of individual genes in each functional cluster are available in Table S1.

Affymetrix gene chip array analyses of spinal cord RNA were initially performed in two control mice and three mice in the RSA59 infected group. However, the data presented in Figure 1A includes just two control mice and two RSA59 infected mice because in the RSA59 infected group one mouse did not get an effective inoculation as demonstrated by histopthological data as well as real time PCR data analysis. Affymetrix gene chip array analysis of spinal cord RNA reveals differential expression of 822 genes in RSA59-infected mice using a 1.5-fold cut off for changes in expression and a P value ≤0.05 (Figure1A) as compared to control mice. Volcano plot of genes expressed in two control and two infected mice reveals that out of these 822 genes, 681 genes were upregulated and 141 genes were down regulated in infected mice. Using a 2-fold cut for significant changes in transcript expression with respect to control, 493 genes were upregulated and only 41 genes were down regulated (data not shown). For remaining studies, we only analyzed the 1.5-fold cut off data. Affymetrix Expression Console Software was used to identify specific upregulated and down regulated (Figure 1B) genes. The down regulated genes mostly belong to non-coding regions of the mouse genome. The 681 genes upregulated in RSA59 infected spinal cords include numerous genes involved in innate immune inflammation, and can be clustered into groups based on their function (Figure1C). Among these functional groups, we identified which genes were most upregulated (4-fold increase and above) and previously not known to be associated with MHV infection and inflammation. These genes are shown in Table 2.

**Table 2:** List of upregulated genes previously not known to be associated with MHV infection and inflammation.

Gene Group	Gene Symbol	Fold Change
Microglia Specific Genes	Aif1	4.141
	Mpeg1	4.32
Guanylate Binding Proteins	GBP1	7.116
	GBP2	29.653
	GBP3	16.972
	GBP4	10.455
	GBP5	9.826
	GBP6	12.627
	GBP8	9.426
	GBP10	27.316
GTPases	Gvin1	6.266–6.986
Interferon Activated and Induced Genes	ifi204	10.786
	ifi205	5.784
	ifi27	14.414
	ifi30	5.789
	iigp1	31.259
	IRGM1	17.216
	IRGM2	28.333
NLR Card Domain	nlrc5	7.803–9.208
T cell specific GTPase	Tgtp1	28.285–38.589

Detailed differential expression levels (fold changes) of individual genes are presented in Table S1. Data reveals that expression of inflammatory mediators like CXCL10, CXCL9, CCL 5 and CCL12 is upregulated. RSA59 infection of the mouse spinal cord is further marked by increased expression of genes involved in IFN signaling including immunity related GTPase (Irgm2|Igtp, Irgm1|Igtp), Interferon inducible protein (Guanylate binding protein; GBP2, GBP3, GBP4, GBP6, GBP8, GBP9), macrophage activation 2 like protein (GBP10), interferon inducible GTPase (IiGP1), and T cell related GTPase. MHC-Class–II, Irf1, Irf7 and Stat1 are also significantly upregulated in the spinal cord of RSA59 infected mice compared to mock-infected controls. Conventional T cell (CD4, CD8) and B cell (CD19) transcripts also show upregulation in microarray analysis, and there is significant upregulation of CD74, CD11b (ITGAM) and CD52, known cell surface markers for microglia/monocytes (Figure 1C and Table S1).

**Figure 2:**
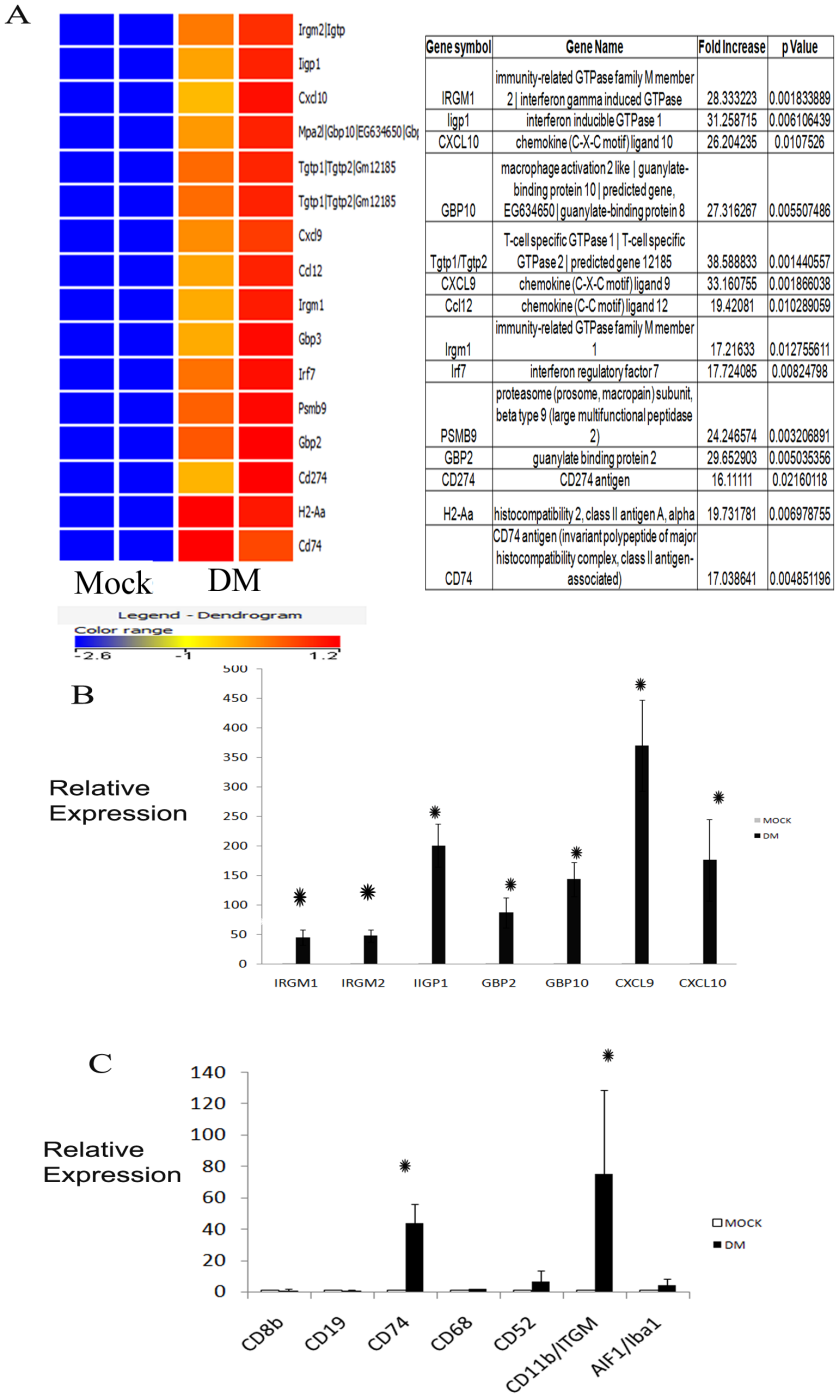
Heat map of the highest expressing innate immunity genes (A). 16 genes are highly up-regulated (16-to 38-fold) as compared to mock-infected control. The color coding dendrogram represents the expression of individual genes which increases from blue (lowest expression) to red (highest expression). Validation of Affymetrix Microarray Data by Real Time PCR Analysis (B and C). Relative mRNA expressions of some of the most highly up-regulated genes are quantified by real time PCR to validate data obtained in Microarray Analysis. The genes selected are immunity related GTPases (IRGM1, IRGM2), guanylate binding proteins (GBP2, GBP10) and Interferon-gamma inducible GTPase 1 (IIGP1), CXCL9 and CXCL10 (B); and CD molecules including CD8b, CD19, CD11b, CD74, CD68, CD52 and Iba1 protein coding gene Aif1 are also validated (C). Data shows that IRGM1, IRGM2, GBP2, GBP10 and IIGP1 are highly up-regulated. Microglia specific CD molecules CD74, CD11b and Aif1 (*Represents significant upregulation. P value of Aif1 <0.005) are also enhanced. DM signifies an average of two RSA59 infected mice. (*Represents significant upregulation. P values of IRGM1 <0.004, IRGM2 <0.003, IIGP1 <0.004, GBP2 <0.0041, GBP10 <0.0023, CD74 <0.002 and CD11b <0.005).

From the list of differentially expressed innate immunity genes, we targeted 16 genes (16–40-fold upregulated) for mechanistic pathways analysis. A heat map of these 16 genes from microarray data is shown in Figure 2A.

### Validation of some of the innate immunity related genes expressed in Microarray Analysis

To validate microarray data, quantitative RT-PCR was performed using primer/probe (Table 1) for key upregulated innate immunity genes (Figure 2B and 2C). β-actin primer was used as internal control. Samples were analyzed in triplicate. Validation was performed for CD molecules, CXCL, IRGM and GBP genes; IFNγ induced genes IRGM1, IRGM2, GBP2, GBP10, Iigp1; and CXCL molecules CXCL9 and CXCL10 which were highly upregulated (Figure 2B). There was no upregulation of CD4 (data not shown) or CD19 (0.9905), and little expression of CD8a and CD8b which is also associated with upregulation of granzyme; thus, despite upregulation of these conventional T and B cell markers by microarray analysis, it is not clear whether they are truly upregulated. There was significant upregulation of microglia-associated CDs, including CD74, CD52, and CD11b (ITGAM). Allograft inflammatory factor 1 gene (Aif1; 4.563), which encodes Iba-1, a calcium-binding protein upregulated during microglia/macrophage activation and phagocytosis, was also upregulated (Figure 2C). Overall, RT-PCR validated microarray data for all upregulated genes examined, except for the conventional T and B cell markers.

### Classical Innate signaling pathway activated in RSA59 infection

**Figure 3:**
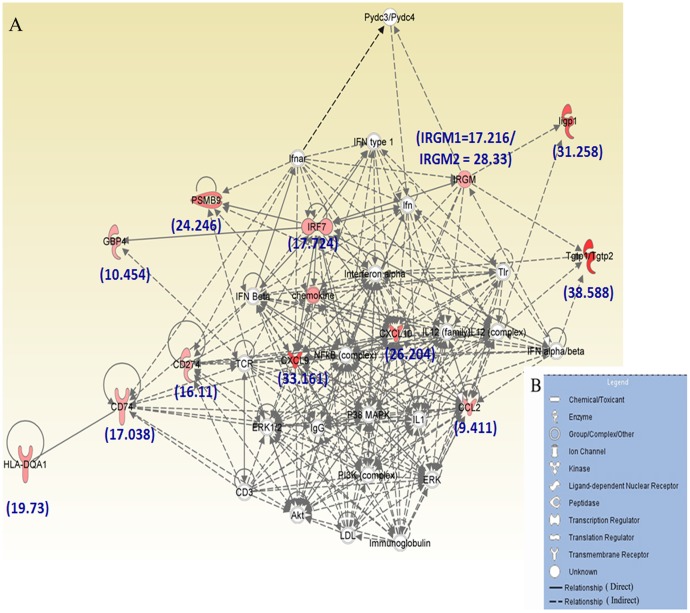
Ingenuity Pathway Analysis (IPA) of the 16 genes most highly up-regulated in microarray analysis. A, Functional network showing the direct and indirect relationships that exist between these molecules which lead to development of immunological diseases. The figure legend (B) shows the different symbols that have been used in the functional network (A).

Biological network and functional pathways analysis of the targeted 16 genes performed using Ingenuity Pathway Analysis (IPA) 8.0 software revealed that most differentially expressed genes are involved in classical innate immune signaling (Figure 3A). The figure legend (B) shows the different symbols that have been used in the functional network (A). IPA analysis identified top molecules involved in known disease pathways including Tgtp1 (38.533 fold increase), CXCL9 (33.161), Iigp1 (31.259), CXCL10 (26.204), PSMB9 (24.247), HLA-DQA1 (19.732), CCL2 (19.421), IRF7 (17.724), and IRGM1/IRGM2 (17.216/28.333). These molecules influence canonical pathways like Antigen Presentation Pathway, multiple sclerosis, Hypercytokinemia/Hyperchemokinemia, granulocyte and Agranulocyte adhesion and diapedesis. The top disease pathways that are influenced by these genes are endocrine system disorders, gastrointestinal and immunological diseases, metabolic disorders and infectious diseases. IPA analysis also identified five molecules (ifnar, ifng, TRIM24, STAT1 and ifnb1) that may play important roles upstream of these canonical pathways.

### RSA59 activates the classical M1 microglial activating pathway

**Figure 4:**
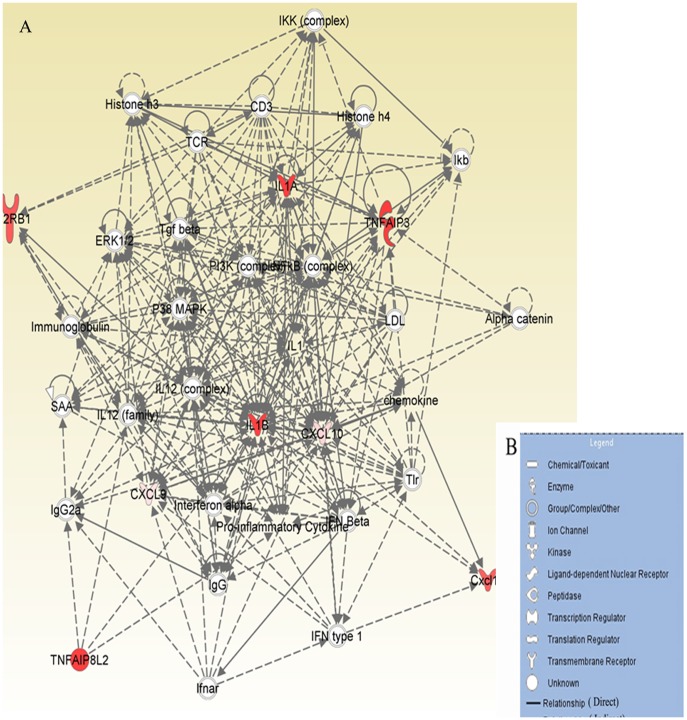
Ingenuity Pathway Analysis (IPA) of the genes involved in M1 microglia/macrophage activation pathways. Functional network (A) showing the direct and indirect relationships that exist between these molecules which lead to myelin loss. The legends of the symbols used in the network are given in (B).

Biological network and functional pathways analysis of the targeted 16 genes also reveals that some of the differentially expressed genes may be involved in activation of classical M1 activation pathways (Figure 4A). The figure legend (B) shows the different symbols that have been used in the functional network (A).The top molecules involved in M1 microglial activation pathways are chemokines (CXCL9, CXCL10 and CXCL11), TNFAIP3, IL-1A, IL-1B, and IL-12rb1. These molecules can also affect various canonical pathways, including multiple sclerosis, Hypercytokinemia/Hyperchemokinemia, granulocyte and Agranulocyte adhesion, diapedesis and communication pathways linking innate and adaptive immune systems. Upstream molecules that are increased include interferons (IFNAR1 and IFNLR1), TBK1 and CXCL10. CXCL10, produced by infected cells and astrocytes, can bind to CXCR3 expressing microglia in the demyelinating plaque while CCL2 can induce the recruitment of microglia in MS lesions. The top disease pathways influenced include inflammatory responses, infectious and hematological diseases, respiratory diseases and skeletal and muscular disorders.

### Immunohistochemical evidence of activation of resting microglia to phagocytotic microglia in RSA59 inflamed brain

**Figure 5:**
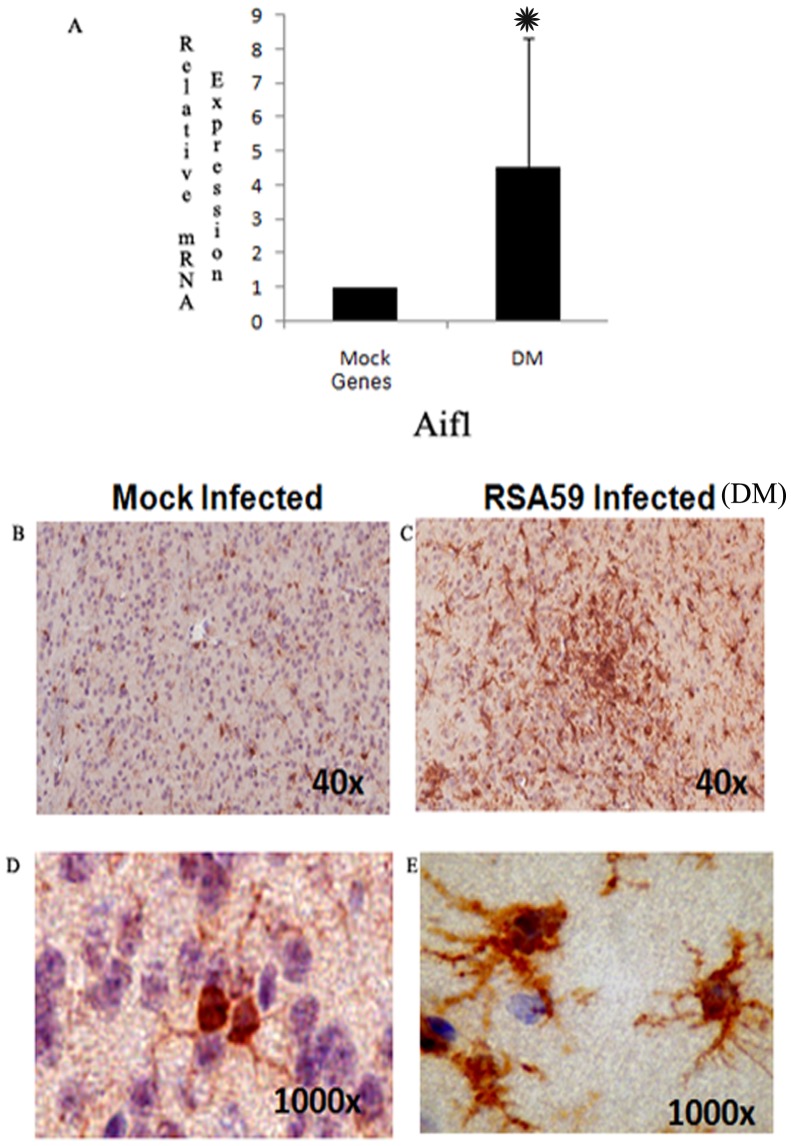
Relative Iba1 (Aif1) mRNA expression and protein expression in RSA59 (DM strain) infected mice compared to mock infected control. Real Time PCR analysis of the Aif1 gene expression showed that there is approximately four fold up regulation in RSA59 infected mice brain compared to mock infection (*Represents significant upregulation. P value of Aif1 <0.005) (A). Immunohistochemistry with anti Iba1 antibody showed a huge upregulation of microglia specific Iba1 protein expression in RSA59 infected brain section (C) compared to mock infected control (B). Higher magnification (1000x) of individual microglia in RSA59 infection showed the presence of large cell body and amoeboid processes (phagocytic in nature) (E). In resting control brain microglia has thin ramified processes and cell size in comparatively smaller. (D).

More detailed immunohistochemical analysis of RSA59 infected brain revealed that there are several nodule formations in inflamed regions like basal forebrain, anterior commissure basal pons, midbrain and deep cerebellar white matter at day 6 post-inoculation. Nodules surrounding inflamed regions stain positive for Iba-1 (microglia marker) and inflammatory responses include changes in microglia morphology-from ramified (resting) to amoeboid (active) (Figure 5C and E), which suggests transformation of resting microglia to phagocytotic microglia. In the mock infected brains, most Iba-1 stained cells show ramified morphology characteristic of resting microglia (Figure 5B and D). Very few cells in the vicinity of the inflamed regions are CD4+, CD8+ or CD19+ (data not shown) as seen in previous studies [10], [15]. Data is consistent with mRNA expression by RT- PCR showing a 4- to 5-fold upregulation of the Aif1 gene which encodes Iba-1 protein (Figure 5C).

### Upregulation of IFN-γ, IL-12, IL-10 and mKC protein expression in acute inflamed spinal cord tissues of RSA59 infected mice

**Figure 6:**
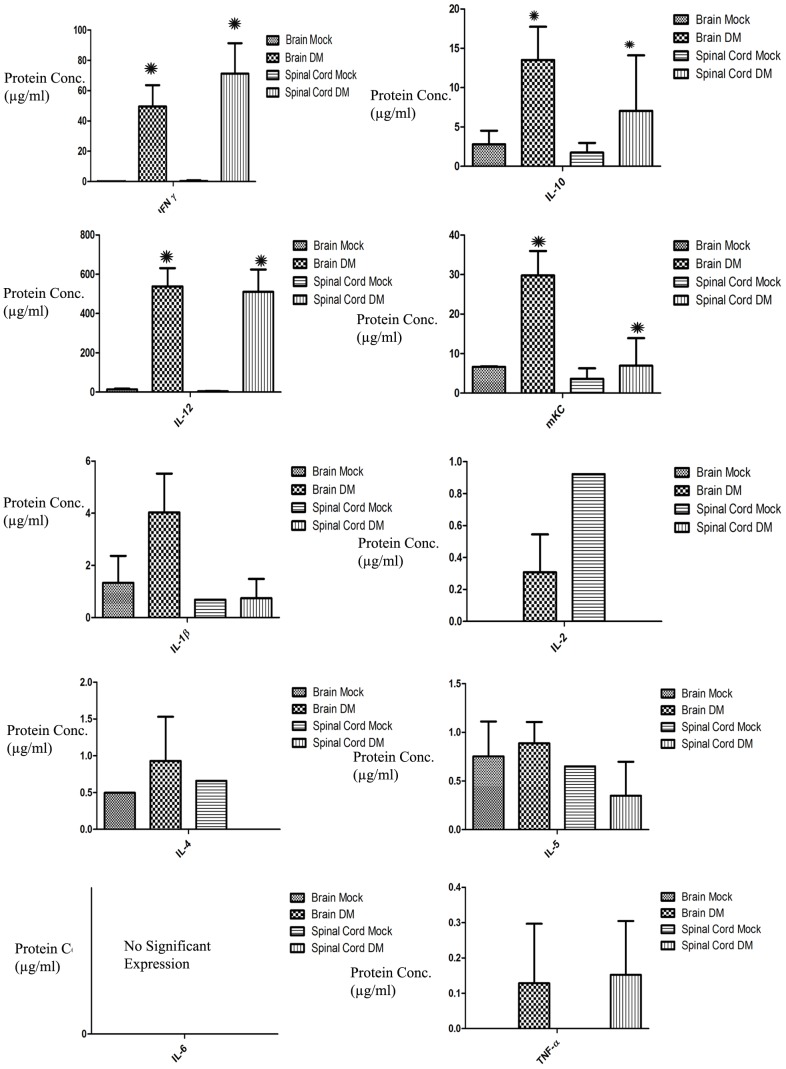
Mouse multiplex array cytokine assay was performed on protein extracts from acute inflamed (day 6 post-infection) brain and spinal cord tissues from RSA59-infected and mock-infected mice. Expression levels of pro- and anti-inflammatory cytokines reveals up-regulation of proteins involved in innate immune responses, including IFN-γ, IL-12, IL-10 and mKC, similar to gene expression data. (* Represents significant upregulation. P values for IFN-γ is <0.002, for IL-10 is <0.005, for IL-12 is <0.004 and for mKC is <0.005).

To determine protein levels of pro-and anti-inflammatory cytokines, sandwich immunoarray-based high throughput MSD96 well Multi-Array and Multispot mouse cytokine assay was performed for IFN-γ, IL-1β, IL-2, IL-4, IL-5, IL-6, mKC/GRO, IL-10, IL-12, and TNF-α. Analysis of acute inflamed brain and spinal cord tissue from RSA59-infected mice reveals robust IFN-γ, IL-12, IL-10 and mKC protein expression (Figure 6). Data corroborates microarray data, showing upregulation of innate immune related proteins mainly involved in antiviral immune responses and phagolysosome maturation, such as IFN-γ and IL-12, which stimulates IFN-γ secretion. mKC is produced by activated microglia/macrophages and plays an important role in inflammation. IL-10 is also upregulated, and is a potent suppressor of monocyte/macrophage function in secretion of TH1 cytokines, and helps virus evade the immune system. Results are consistent with prior evidence that a Th1-biased cytokine/chemokine response accompanies persistent MHV infection when antigen presentation is ongoing [16].

## Discussion

The current mRNA profiling data demonstrates significant upregulation of genes involved in innate immune responses during acute MHV infection. Results are not unexpected, as they are consistent with histopathological data demonstrating the activation of microglia and innate immune response in CNS tissues following intracranial inoculation. Results are also consistent with prior studies that have examined the role of specific proteins, including some of the cytokines, chemokines and signaling molecules whose gene expression was found to be upregulated here. In addition, the current approaches also identified a number of other genes that were upregulated in MHV induced neuroinflammation but were not reported before, and thus warrant further study to explore their role in MHV infection in CNS cells.

Interestingly, our studies did not find significant changes in genes involved in lymphocytic activation, with upregulation of T and B cell markers on microarray failing to be validated by RT-PCR. Previously, it was shown that that RAG1-/- mice on B6 background infected with RSA59 develop fatal disease but not WT B6 mice, which supports the importance of adaptive immune response in this disease [17]. But the fatal disease of RSA59 in RAG1-/- mice may also be due to hypersensitivity. Moreover, there is evidence that MHV-A59 (Parental wild type strain of RSA59) can induce demyelination in the absence of T and B cells in RAG1-/- mice [18]. Taken together with our current findings, one possible explanation is that RSA59 may induce virus specific CD4 and CD8 T cells at day 7 p.i., but at earlier days (day 3 through 6); the majority of the inflammatory cells are microglia. Direct virus induced neuroinflammation due to the activation of CNS resident microglia may induce virus-specific CD8 and CD4 T cell responses in the CNS which may play a role in the augmentation of inflammation. Therefore, our studies mainly found changes in genes which are known to code microglia/macrophage specific cell surface molecules and other features of the innate response, often felt to occur even earlier in acute viral infection.

Many of the genes undergoing significant upregulation in the current study encode factors that have previously been shown to play a role in a variety of acute viral infections. It is well known that Type I interferons (IFN-α/β) provide a first line of defense against viral infections by limiting dissemination prior to the emergence of adaptive immune responses. Viral infection is sensed by endosomal Toll-like receptors (TLRs), which signal to activate NF-κB, IFN-regulatory factor 3 (IRF3), IRF7, and downstream transcription of cytokine and IFN-α/β genes [19], [20]. Previous studies demonstrated that IFN-α/β binding to its receptor induces JAK/STAT signaling and upregulation of hundreds of IFN-stimulated genes (ISGs) in infected and uninfected cells. The antiviral effector functions triggered by IFN-α/β during a virus infection involve the concerted action of ISGs. In addition to direct innate antiviral factors, ISGs comprise cytokines, chemokines, and molecules associated with antigen presentation (MHC class I), thereby shaping adaptive immune responses [21]. The classically studied ISGs, RNase L, PKR, and Mx1, disrupt viral replication and subsequently spread by interfering with both viral and host cell transcription and translation [22]. However, the susceptibility to innate antiviral effector mechanisms differs between viruses and may also be distinct between target organs as well as cell types and their differentiation state [23], [24] has been reported.

CXCL9 and CXCL10 are known to provide antiviral immune response in MHV infection. Insertion of CXC chemokine ligands 9 into the MHV genome resulted in protection from viral induced encephalitis and hepatitis [25], [26]. The IFN-induced proteins with tetratricopeptide repeats (IFIT) are expressed in very low basal levels but are the most strongly induced ISGs during many viral infections [27], [28], [29], [30]. One recent study demonstrated that IFIT2 deficiency results in uncontrolled coronavirus replication and enhanced encephalitis via impaired IFN-α/β induction in macrophages [31].

Our current studies also found the alteration of different innate immune response genes involved in IFN activation pathways, cytokine/chemokine activation pathways, endosomal Toll like receptor activation pathways, IRF, and Jack/Stat pathways, similar to changes noted in the studies mentioned above. But our current studies also found upregulation of additional molecules previously unknown to have a role (Table 2), including microglia specific genes, Guanylate Binding Proteins, GTPases, Interferon activated and induced genes, NLR Card Domain – 5, and T cell specific GTPase. The role of individual genes in reducing viral replication and innate immunity has yet to be characterized. It will be interesting to explore the role of ISGs in microglia activation and phagocytosis. Moreover, their contribution to protection in individual cell types and tissues *in vivo* warrants further studies. In general, understanding the role of these genes may enhance the knowledge of innate immunity against neurotropic viruses and its role in shaping the adaptive immune responses.

The role of microglial activation observed in early MHV infection is similar to its importance in other neurotropic virus infections, such as HIV induced dementia (HAD) [32], [33], [34], Cytomegalovirus infection [35], [36], Herpes Simplex Virus (HSV) infection [37], [38], as well as in other neurodegenerative diseases like Alzheimer's [39], [40], [41]. HIV-1 enters the CNS early after infection, whereas productive replication and macrophage invasion occur years later [42]. Infected microglia harbor viral particles intracellularly, reflecting their potential as a reservoir. It has become increasingly apparent that HIV-1-infected microglia actively secrete both endogenous neurotoxins (TNF-α, IL-1α, CXCL8/IL-8, glutamate, quinolinic acid, platelet-activating factor, eicosanoids, and nitric oxide) as well as neurotoxic viral proteins (Tat, gp120, and gp41). In addition to neurotoxicity, these viral proteins can also affect microglial cell proliferation [42]. In Cytomegalovirus infection (HCMV), key defence cells include microglia and T lymphocytes. In mouse models, antiviral cytokine TNF-α is produced by the microglial cells and sometimes T lymphocytes, mainly when they are chemoattracted by CXCL10. In HSV infection, neuroinflammation is characterized by acute focal necrotizing encephalitis. Microglial cells express both MHC class I and class II molecules and produce soluble mediators TNFα, IL-1α, CXCL10, CCL5, IL-6, CXCL8 and CCL3 [42].

These secreted chemokines may induce neuronal death directly by activating neuronal chemokine receptors or indirectly by the activation of microglia mediated autophagic mechanisms [43]. Thus, the activation of microglia and related upregulation of genes expressed by activated microglia observed in acute MHV infection is consistent with conserved mechanisms of viral mediated pathology in other infections, and may be less specific for demyelinating disease itself.

Protein expression data corroborates microarray data, including an upregulation of specific Th1 immune related proteins. Many of these proteins are mainly involved in antiviral immune responses and phagolysosome maturation, and their upregulation also suggests acute MHV infection can drive innate and conserved antiviral responses similar to those seen in other viral infections. Similar phenomena have been observed in HIV infection in monocytes/microglia [44]. In contrast to Th1 responses, current results show almost no upregulation of Th2 cytokines/chemokines. The combination of increased expression of several canonical Th1 cytokines/chemokines and absence of Th2 cytokines/chemokines in RSA59 infected tissues supports the hypothesis that the host is continuing to mount a cell-mediated immune response to RSA59, as previously suggested [45]. Moreover, our data suggest that activated microglia/macrophage and inflammatory mediators contribute to a local CNS microenvironment that regulates viral replication and IFN-γ production during the acute phase of infection. The potential role of IFN-γ despite the limited role of T cells is intriguing, as initially it was believed that CD4+ T helper cell type 1 (Th1) lymphocytes, CD8+ cytotoxic lymphocytes, and NK cells exclusively produced IFN- γ [46], [47]. However, there is now evidence that other cells, such as B cells, NKT cells, and professional antigen-presenting cells (APCs) [monocyte/macrophage, dendritic cells (DCs)] secrete IFN-γ [48], [49], [50], [51]. IFN-γ production by monocyte/macrophage, DCs acting locally may be important in self-activation and activation of nearby cells [50], [51]. While IFN γ is produced in T cells, its upregulation has also occurred in T cell deficient nude mice, leading to the conclusion that macrophages can also play a role in this in vivo response of IFN-γ secretion [52], [53], and during viral infections, macrophages are among the first cells in any organ to be exposed and are likely the major producers of IFN-γ soon after infection.

IFN-γ can further self-activate microglia, promoting phagolysome maturation and phagocytosis of the myelin sheath, which may partially explain how demyelination, which starts as early as day 5 post-inoculation and reaches its peak around day 30 [54], occurs. Previous ultrastructural studies [10] support this mechanism as well, revealing activated microglia can surround myelinated axons with the myelin sheath getting disrupted with intact axons. Therefore, one mechanism of demyelination involves macrophage-mediated myelin stripping through an autophagy pathway without involving conventional CD4+ and CD8+T cells.

## Supporting Information

Figure S1 Representative histopathology and immunohistochemical analysis of RSA59- and mock-infected mouse brain and liver. Brain tissue from the infected mice whose spinal cord tissues were processed for affymetrix microarray analysis was processed for routine histological and immunohistochemical analysis. A, C, E and G sections are from mock-infected mouse; B, D, F and H are from representative image of RSA59 infected mouse; A–B: liver tissues stained with H & E show severe hepatitis in RSA59 infected mice (B) and no hepatitis lesions in control mice (A). Sagittal brain sections stained with H& E (C, D), immunostained with anti-Iba-1 (E, F), and with anti-nucleocapsid antibody (G–H). RSA59 infected mice show encephalitis (D), diffused staining of Iba-1 (F) and wide spread distribution of viral antigen (H). Representative section from one infected mice were shown here. In control mock infected mice there were no encephalitis was observed (C), resting ramified microglia was observed in E and there were no viral antigen staining as expected (G). I: Relative mRNA expression of viral nucleocapsid gene from two RSA59- (DM) infected mouse spinal cords at day 6 post-infection compared to two control mock-infected mouse spinal cords. Y-axis represents the relative expression of viral nucleocapsid gene of infected and control mice spinal cord and X-axis represents individual mouse from control and infected group.
(TIF)

Table S1 Differential expressed genes between control and RSA59 infected mice were clustered based on their function. Classification of differential expressed genes in the spinal cord between control and RSA59 infected mice.
(DOC)

## References

[1] Streit WJ, Mrak RE, Griffin WS (2004) Microglia and neuroinflammation: a pathological perspective. J Neuroinflammation 1: 14. 15285801 10.1186/1742-2094-1-14 PMC509427

[2] Chatterjee D, Biswas K, Nag S, Ramachandra SG, Das Sarma J (2013) Microglia play a major role in direct viral-induced demyelination. Clin Dev Immunol 2013: 510396. 23864878 10.1155/2013/510396 PMC3705805

[3] Das Sarma J, Scheen E, Seo S-H, Koval M, Weiss SR (2002) Enhanced green fluorescent protein expression may be used to monitor murine coronavirus spread in vitro and in the mouse central nervous system. Journal of Neurovirology 8: 381–391. 12402164 10.1080/13550280260422686 PMC7095158

[4] Das Sarma J, Iacono K, Gard L, Marek R, Kenyon LC, et al (2008) Demyelinating and nondemyelinating strains of mouse hepatitis virus differ in their neural cell tropism. Journal of Virology 82: 5519–5526. 18385249 10.1128/JVI.01488-07 PMC2395180

[5] Gallagher TM, Buchmeier MJ (2001) Coronavirus spike proteins in viral entry and pathogenesis. Virology 279: 371–374. 11162792 10.1006/viro.2000.0757 PMC7133764

[6] Kuo L, Godeke GJ, Raamsman MJ, Masters PS, Rottier PJ (2000) Retargeting of coronavirus by substitution of the spike glycoprotein ectodomain: crossing the host cell species barrier. J Virol 74: 1393–1406. 10627550 10.1128/jvi.74.3.1393-1406.2000 PMC111474

[7] Das Sarma J, Fu L, Tsai JC, Weiss SR, Lavi E (2000) Demyelination determinants map to the spike glycoprotein gene of coronavirus mouse hepatitis virus. J Virol 74: 9206–9213. 10982367 10.1128/jvi.74.19.9206-9213.2000 PMC102119

[8] Phillips JJ, Chua MM, Lavi E, Weiss SR (1999) Pathogenesis of chimeric MHV4/MHV-A59 recombinant viruses: the murine coronavirus spike protein is a major determinant of neurovirulence. J Virol 73: 7752–7760. 10438865 10.1128/jvi.73.9.7752-7760.1999 PMC104302

[9] Phillips JJ, Chua M, Seo SH, Weiss SR (2001) Multiple regions of the murine coronavirus spike glycoprotein influence neurovirulence. J Neurovirol 7: 421–431. 11582514 10.1080/135502801753170273 PMC7095106

[10] Das Sarma J, Kenyon LC, Hingley ST, Shindler KS (2009) Mechanisms of primary axonal damage in a viral model of multiple sclerosis. J Neurosci 29: 10272–10280. 19692601 10.1523/JNEUROSCI.1975-09.2009 PMC2747667

[11] Das Sarma J, Chatterjee K, Dalui T, Ghosh S (2013) Tissue Specific Optimization Of Haematoxylin and Eosin Stain: An Experiment Accomplished By Varying The Period Of Fixation And Duration Of Stain. Online Journal of Biosciences and Informatics 4: 64–81.

[12] Das Sarma S, Chatterjee K, Dinda H, Chatterjee D, Das Sarma J (2013) Cytomorphological and Cytochemical Identification of Microglia ISRN Immunology. 2013: 10.

[13] Das Sarma J (2014) Microglia-mediated neuroinflammation is an amplifier of virus-induced neuropathology. J Neurovirol 20: 122–136. 23979705 10.1007/s13365-013-0188-4

[14] Das Sarma J (2010) A mechanism of virus-induced demyelination. Interdiscip Perspect Infect Dis 2010: 109239. 20652053 10.1155/2010/109239 PMC2905936

[15] Shindler KS, Chatterjee D, Biswas K, Goyal A, Dutt M, et al (2011) Macrophage-mediated optic neuritis induced by retrograde axonal transport of spike gene recombinant mouse hepatitis virus. J Neuropathol Exp Neurol 70: 470–480. 21572336 10.1097/NEN.0b013e31821da499 PMC3110774

[16] Elliott R, Li F, Dragomir I, Chua MM, Gregory BD, et al (2013) Analysis of the host transcriptome from demyelinating spinal cord of murine coronavirus-infected mice. PLoS One 8: e75346. 24058676 10.1371/journal.pone.0075346 PMC3776850

[17] Phillips JJ, Chua MM, Rall GF, Weiss SR (2002) Murine coronavirus spike glycoprotein mediates degree of viral spread, inflammation, and virus-induced immunopathology in the central nervous system. Virology 301: 109–120. 12359451 10.1006/viro.2002.1551 PMC7131834

[18] Matthews AE, Lavi E, Weiss SR, Paterson Y (2002) Neither B cells nor T cells are required for CNS demyelination in mice persistently infected with MHV-A59. J Neurovirol 8: 257–264. 12053280 10.1080/13550280290049697 PMC7095043

[19] Kumar H, Kawai T, Akira S (2009) Pathogen recognition in the innate immune response. Biochem J 420: 1–16. 19382893 10.1042/BJ20090272

[20] Wilkins C, Gale M Jr (2010) Recognition of viruses by cytoplasmic sensors. Curr Opin Immunol 22: 41–47. 20061127 10.1016/j.coi.2009.12.003 PMC3172156

[21] Samuel CE (2001) Antiviral actions of interferons. Clin Microbiol Rev 14: 778–809, table of contents. 10.1128/CMR.14.4.778-809.2001 PMC89003 11585785

[22] Schoggins JW, Rice CM (2011) Interferon-stimulated genes and their antiviral effector functions. Curr Opin Virol 1: 519–525. 22328912 10.1016/j.coviro.2011.10.008 PMC3274382

[23] Cho H, Proll SC, Szretter KJ, Katze MG, Gale M Jr, et al (2013) Differential innate immune response programs in neuronal subtypes determine susceptibility to infection in the brain by positive-stranded RNA viruses. Nat Med 19: 458–464. 23455712 10.1038/nm.3108 PMC3618596

[24] Zhao L, Birdwell LD, Wu A, Elliott R, Rose KM, et al (2013) Cell-type-specific activation of the oligoadenylate synthetase-RNase L pathway by a murine coronavirus. J Virol 87: 8408–8418. 23698313 10.1128/JVI.00769-13 PMC3719824

[25] Lane TE, Asensio VC, Yu N, Paoletti AD, Campbell IL, et al (1998) Dynamic regulation of alpha- and beta-chemokine expression in the central nervous system during mouse hepatitis virus-induced demyelinating disease. J Immunol 160: 970–978. 9551936

[26] Muse M, Kane JA, Carr DJ, Farber JM, Lane TE (2008) Insertion of the CXC chemokine ligand 9 (CXCL9) into the mouse hepatitis virus genome results in protection from viral-induced encephalitis and hepatitis. Virology 382: 132–144. 18973912 10.1016/j.virol.2008.09.032 PMC2643215

[27] Diamond MS, Farzan M (2013) The broad-spectrum antiviral functions of IFIT and IFITM proteins. Nat Rev Immunol 13: 46–57. 23237964 10.1038/nri3344 PMC3773942

[28] Fensterl V, Sen GC (2011) The ISG56/IFIT1 gene family. J Interferon Cytokine Res 31: 71–78. 20950130 10.1089/jir.2010.0101 PMC3021354

[29] Terenzi F, Pal S, Sen GC (2005) Induction and mode of action of the viral stress-inducible murine proteins, P56 and P54. Virology 340: 116–124. 16023166 10.1016/j.virol.2005.06.011

[30] Wacher C, Muller M, Hofer MJ, Getts DR, Zabaras R, et al (2007) Coordinated regulation and widespread cellular expression of interferon-stimulated genes (ISG) ISG-49, ISG-54, and ISG-56 in the central nervous system after infection with distinct viruses. J Virol 81: 860–871. 17079283 10.1128/JVI.01167-06 PMC1797448

[31] Butchi NB, Hinton DR, Stohlman SA, Kapil P, Fensterl V, et al (2014) Ifit2 deficiency results in uncontrolled neurotropic coronavirus replication and enhanced encephalitis via impaired alpha/beta interferon induction in macrophages. J Virol 88: 1051–1064. 24198415 10.1128/JVI.02272-13 PMC3911674

[32] Kaul M, Garden GA, Lipton SA (2001) Pathways to neuronal injury and apoptosis in HIV-associated dementia. Nature 410: 988–994. 11309629 10.1038/35073667

[33] Minagar A, Shapshak P, Fujimura R, Ownby R, Heyes M, et al (2002) The role of macrophage/microglia and astrocytes in the pathogenesis of three neurologic disorders: HIV-associated dementia, Alzheimer disease, and multiple sclerosis. J Neurol Sci 202: 13–23. 12220687 10.1016/s0022-510x(02)00207-1

[34] Bell JE (1998) The neuropathology of adult HIV infection. Rev Neurol (Paris) 154: 816–829. 9932303

[35] Cheeran MC, Hu S, Sheng WS, Peterson PK, Lokensgard JR (2003) CXCL10 production from cytomegalovirus-stimulated microglia is regulated by both human and viral interleukin-10. J Virol 77: 4502–4515. 12663757 10.1128/JVI.77.8.4502-4515.2003 PMC152158

[36] Cheeran MC, Hu S, Yager SL, Gekker G, Peterson PK, et al (2001) Cytomegalovirus induces cytokine and chemokine production differentially in microglia and astrocytes: antiviral implications. J Neurovirol 7: 135–147. 11517386 10.1080/13550280152058799

[37] Lokensgard JR, Hu S, Sheng W, vanOijen M, Cox D, et al (2001) Robust expression of TNF-alpha, IL-1beta, RANTES, and IP-10 by human microglial cells during nonproductive infection with herpes simplex virus. J Neurovirol 7: 208–219. 11517395 10.1080/13550280152403254

[38] Marques CP, Cheeran MC, Palmquist JM, Hu S, Urban SL, et al (2008) Prolonged microglial cell activation and lymphocyte infiltration following experimental herpes encephalitis. J Immunol 181: 6417–6426. 18941232 10.4049/jimmunol.181.9.6417 PMC2614272

[39] Versijpt JJ, Dumont F, Van Laere KJ, Decoo D, Santens P, et al (2003) Assessment of neuroinflammation and microglial activation in Alzheimer's disease with radiolabelled PK11195 and single photon emission computed tomography. A pilot study. Eur Neurol 50: 39–47. 12824711 10.1159/000070857

[40] Streit WJ (2010) Microglial activation and neuroinflammation in Alzheimer's disease: a critical examination of recent history. Front Aging Neurosci 2: 22. 20577641 10.3389/fnagi.2010.00022 PMC2890154

[41] Weitz TM, Town T (2012) Microglia in Alzheimer's Disease: It's All About Context. Int J Alzheimers Dis 2012: 314185. 22779026 10.1155/2012/314185 PMC3388286

[42] Rock RB, Gekker G, Hu S, Sheng WS, Cheeran M, et al (2004) Role of microglia in central nervous system infections. Clin Microbiol Rev 17: 942–964 table of contents.. 15489356 10.1128/CMR.17.4.942-964.2004 PMC523558

[43] Cartier L, Hartley O, Dubois-Dauphin M, Krause KH (2005) Chemokine receptors in the central nervous system: role in brain inflammation and neurodegenerative diseases. Brain Res Brain Res Rev 48: 16–42. 15708626 10.1016/j.brainresrev.2004.07.021

[44] Sozzani S, Ghezzi S, Iannolo G, Luini W, Borsatti A, et al (1998) Interleukin 10 increases CCR5 expression and HIV infection in human monocytes. J Exp Med 187: 439–444. 9449724 10.1084/jem.187.3.439 PMC2212126

[45] Chen BP, Kuziel WA, Lane TE (2001) Lack of CCR2 results in increased mortality and impaired leukocyte activation and trafficking following infection of the central nervous system with a neurotropic coronavirus. J Immunol 167: 4585–4592. 11591787 10.4049/jimmunol.167.8.4585

[46] Bach EA, Aguet M, Schreiber RD (1997) The IFN gamma receptor: a paradigm for cytokine receptor signaling. Annu Rev Immunol 15: 563–591. 9143700 10.1146/annurev.immunol.15.1.563

[47] Young HA (1996) Regulation of interferon-gamma gene expression. J Interferon Cytokine Res 16: 563–568. 8877725 10.1089/jir.1996.16.563

[48] Frucht DM, Fukao T, Bogdan C, Schindler H, O'Shea JJ, et al (2001) IFN-gamma production by antigen-presenting cells: mechanisms emerge. Trends Immunol 22: 556–560. 11574279 10.1016/s1471-4906(01)02005-1

[49] Gessani S, Belardelli F (1998) IFN-gamma expression in macrophages and its possible biological significance. Cytokine Growth Factor Rev 9: 117–123. 9754706 10.1016/s1359-6101(98)00007-0

[50] Harris DP, Haynes L, Sayles PC, Duso DK, Eaton SM, et al (2000) Reciprocal regulation of polarized cytokine production by effector B and T cells. Nat Immunol 1: 475–482. 11101868 10.1038/82717

[51] Sen GC (2001) Viruses and interferons. Annu Rev Microbiol 55: 255–281. 11544356 10.1146/annurev.micro.55.1.255

[52] Cockfield SM, Ramassar V, Halloran PF (1993) Regulation of IFN-gamma and tumor necrosis factor-alpha expression in vivo. Effects of cycloheximide and cyclosporine in normal and lipopolysaccharide-treated mice. J Immunol 150: 342–352. 8419467

[53] Mogensen SC, Virelizier JL (1987) The interferon-macrophage alliance. Interferon 8: 55–84. 2445689

[54] Shindler KS, Kenyon LC, Dutt M, Hingley ST, Das Sarma J (2008) Experimental optic neuritis induced by a demyelinating strain of mouse hepatitis virus. J Virol 82: 8882–8886. 18579591 10.1128/JVI.00920-08 PMC2519666

