# Supplementary figures and images for: Mouse Hepatitis Virus Infection Upregulates Genes Involved in Innate Immune Responses
Source: PLoS One. 2014 Oct 31;9(10):e111351. doi: 10.1371/journal.pone.0111351 (PMC4216085; doi:10.1371/journal.pone.0111351)

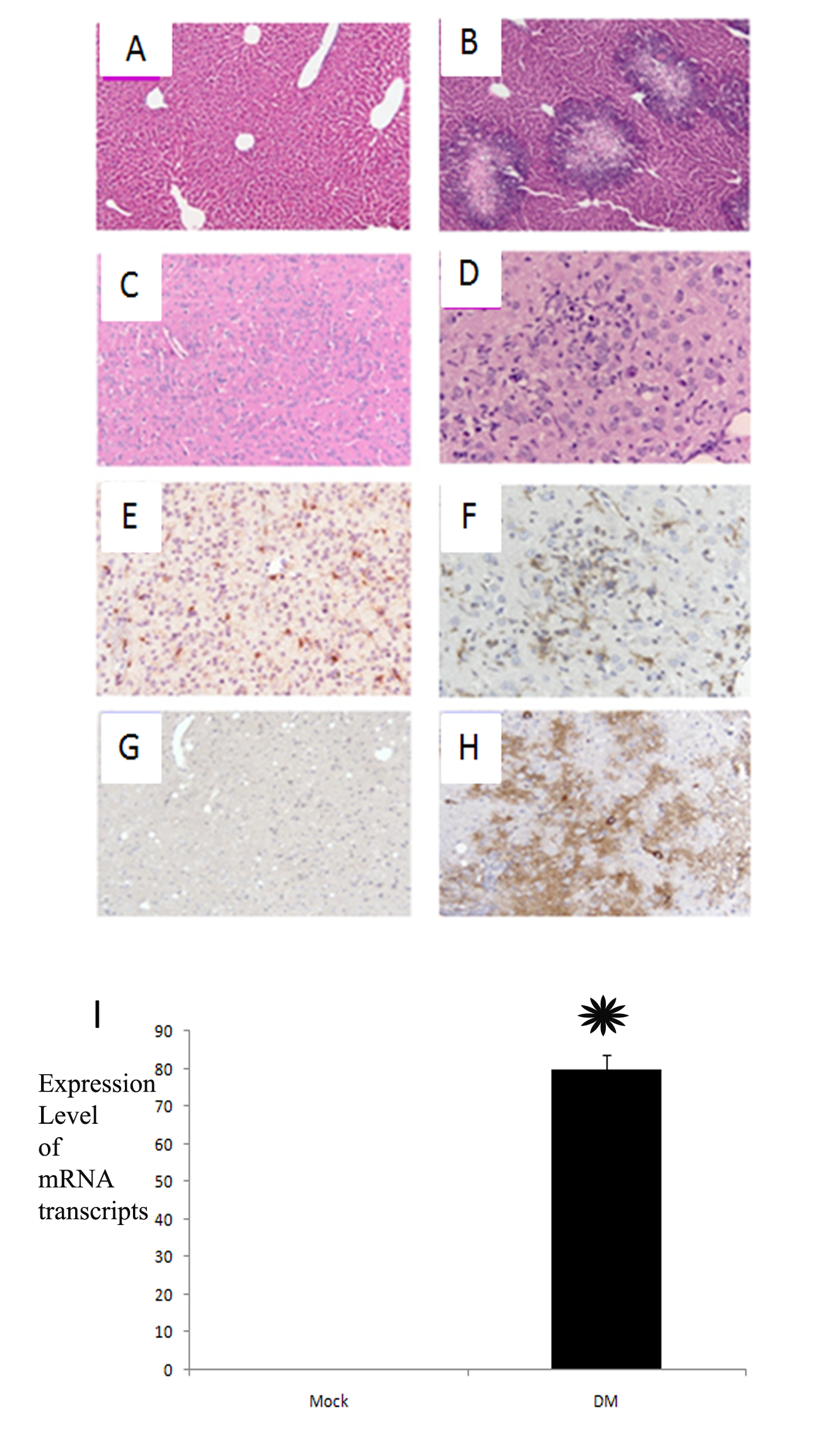

Supplement: Figure S1 — Representative histopathology and immunohistochemical analysis of RSA59- and mock-infected mouse brain and liver. Brain tissue from the infected mice whose spinal cord tissues were processed for affymetrix microarray analysis was processed for routine histological and immunohistochemical analysis. A, C, E and G sections are from mock-infected mouse; B, D, F and H are from representative image of RSA59 infected mouse; A–B: liver tissues stained with H & E show severe hepatitis in RSA59 infected mice (B) and no hepatitis lesions in control mice (A). Sagittal brain sections stained with H& E (C, D), immunostained with anti-Iba-1 (E, F), and with anti-nucleocapsid antibody (G–H). RSA59 infected mice show encephalitis (D), diffused staining of Iba-1 (F) and wide spread distribution of viral antigen (H). Representative section from one infected mice were shown here. In control mock infected mice there were no encephalitis was observed (C), resting ramified microglia was observed in E and there were no viral antigen staining as expected (G). I: Relative mRNA expression of viral nucleocapsid gene from two RSA59- (DM) infected mouse spinal cords at day 6 post-infection compared to two control mock-infected mouse spinal cords. Y-axis represents the relative expression of viral nucleocapsid gene of infected and control mice spinal cord and X-axis represents individual mouse from control and infected group. (TIF) [file pone.0111351.s001.tif]
