# Supplementary material for: Mouse Hepatitis Virus Infection Upregulates Genes Involved in Innate Immune Responses
Source: PLoS One. 2014 Oct 31;9(10):e111351. doi: 10.1371/journal.pone.0111351 (PMC4216085; doi:10.1371/journal.pone.0111351)
Supplement: Table S1 — Differential expressed genes between control and RSA59 infected mice were clustered based on their function. Classification of differential expressed genes in the spinal cord between control and RSA59 infected mice. (DOC) [file pone.0111351.s002.doc]

SUPPLE TABLE 1.

**Classification of Genes Upregulated or Downregulated Based on Their Functions**

| 1. **Apoptosis Related Genes** | | | | | |
| --- | --- | --- | --- | --- | --- |
|  |  |  |  |  |  |
| Transcripts Cluster Id | Gene description | Gene symbol | p value | Fold Change | Regulation |
| 10413229 | annexin A11 | anxa11 | 0.0260 | 1.600 | up |
| 10523451 | annexin A3 | anxa3 | 0.0004 | 2.090 | up |
| 10582985 | caspase 1 | casp1 | 0.0429 | 2.660 | up |
| 10583008 | caspase 12 | casp12 | 0.0199 | 2.680 | up |
| 10537728 | caspase 2 | casp2 | 0.0130 | 1.730 | up |
| 10582997 | caspase 4, apoptosis-related cysteine peptidase | casp4 | 0.0139 | 4.180 | up |
| 10464128 | caspase 7 | casp7 | 0.0483 | 2.475 | up |
| 10346564 | caspase 8 | casp8 | 0.0129 | 2.480 | up |

| 1. **Microglia Specific Genes** | | | | | |
| --- | --- | --- | --- | --- | --- |
|  |  |  |  |  |  |
| Transcripts Cluster Id | Gene description | Gene symbol | p value | Fold Change | Regulation |
| 10450484 | allograft inflammatory factor 1 | aif1 | 0.0353 | 4.141 | up |
| 10461721 | macrophage expressed gene 1 | mpeg1 | 0.0041 | 4.320 | up |
| 10508663 | lysosomal-associated protein transmembrane 5 | laptm5 | 0.0196 | 3.570 | up |
| 10372652 | lysozyme 1 | lyz1 | 0.0057 | 3.159 | up |
| 10372648 | lysozyme 2 | lyz2 | 0.0127 | 4.322 | up |

| 1. **Complement Component** | | | | | |
| --- | --- | --- | --- | --- | --- |
| Transcripts Cluster Id | Gene description | Gene symbol | p value | Fold Change | Regulation |
| 10517517 | complement component 1, q subcomponent, alpha polypeptide | c1qa | 0.0027 | 4.388 | up |
| 10517508 | complement component 1, q subcomponent, beta polypeptide | c1qb | 0.0078 | 3.316 | up |
| 10517513 | complement component 1, q subcomponent, C chain | c1qc | 0.0065 | 2.531 | up |
| 10541678 | complement component 1, r subcomponent A | c1ra | 0.0291 | 2.404 | up |
| 10541683 | complement component 1, r subcomponent B | complement component 1, r subcomponent A | c1rb|c1ra | 0.0248 | 2.755 | up |
| 10541670 | complement component 1, r subcomponent-like | c1rl | 0.0267 | 1.608 | up |
| 10547740 | complement component 1, s subcomponent | c1s | 0.0099 | 2.279 | up |
| 10450344 | complement component 2 (within H-2S) | c2 | 0.0295 | 2.817 | up |
| 10450280 | complement component 4A (Rodgers blood group) | c4a | 0.0023 | 3.445 | up |
| 10450242 | complement component 4B (Childo blood group) | complement component 4A (Rodgers blood group) | c4b|c4a | 0.0011 | 6.086 | up |
| 10480751 | complement component 8, gamma polypeptide | c8g | 0.0279 | -1.888 | down |
| 10363475 | perforin 1 (pore forming protein) | prf1 | 0.0048 | 1.729 | up |

| **4. Chemokine, Cytokine and Ligands** | | | | | |
| --- | --- | --- | --- | --- | --- |
|  |  |  |  |  |  |
| Transcripts Cluster Id | Gene description | Gene symbol | p value | Fold Change | Regulation |
| 10379530 | chemokine (C-C motif) ligand 12 | ccl12 | 0.0102 | 19.420 | up |
| 10504132 | chemokine (C-C motif) ligand 19 | similar to EBI-1 ligand chemokine | ccl19|LOC100043921|LOC100043918 | 0.0496 | 4.677 | up |
| 10504159 | chemokine (C-C motif) ligand 19 | similar to EBI-1 ligand chemokine | ccl19|LOC100043921|LOC100043918 | 0.0461 | 4.676 | up |
| 10504188 | chemokine (C-C motif) ligand 19 | similar to EBI-1 ligand chemokine | ccl19|LOC100043921|LOC100043918 | 0.0463 | 4.679 | up |
| 10512322 | chemokine (C-C motif) ligand 19 | similar to EBI-1 ligand chemokine | ccl19|LOC100043921|LOC100043918 | 0.0461 | 4.681 | up |
| 10512372 | chemokine (C-C motif) ligand 19 | similar to EBI-1 ligand chemokine | ccl19|LOC100043921|LOC100043918 | 0.0461 | 4.680 | up |
| 10379511 | chemokine (C-C motif) ligand 2 | ccl2 | 0.0255 | 9.411 | up |
| 10379721 | chemokine (C-C motif) ligand 4 | ccl4 | 0.0103 | 5.194 | Up |
| 10389207 | chemokine (C-C motif) ligand 5 | ccl5 | 0.0069 | 13.613 | up |
| 10379518 | chemokine (C-C motif) ligand 7 | ccl7 | 0.0146 | 9.798 | up |
| 10379535 | chemokine (C-C motif) ligand 8 | ccl8 | 0.0283 | 2.819 | up |
| 10590631 | chemokine (C-C motif) receptor 2 | ccr2 | 0.0127 | 4.497 | up |
| 10590635 | chemokine (C-C motif) receptor 5 | chemokine (C-C motif) receptor 2 | ccr5|ccr2 | 0.0196 | 3.895 | up |
| 10598013 | chemokine (C-C motif) receptor 5 | chemokine (C-C motif) receptor 2 | ccr5|ccr2 | 0.0194 | 3.879 | up |
| 10532711 | chemokine-like receptor 1 | cmklr1 | 0.0219 | 1.687 | up |
| 10531415 | chemokine (C-X-C motif) ligand 10 | cxcl10 | 0.0108 | 26.204 | up |
| 10531420 | chemokine (C-X-C motif) ligand 11 | cxcl11 | 0.0263 | 7.173 | up |
| 10523359 | chemokine (C-X-C motif) ligand 13 | cxcl13 | 0.0206 | 2.400 | up |
| 10387890 | chemokine (C-X-C motif) ligand 16 | zinc finger, MYND-type containing 15 | cxcl16|zmynd15 | 0.0009 | 3.516 | up |
| 10531407 | chemokine (C-X-C motif) ligand 9 | cxcl9 | 0.0019 | 33.161 | up |
| 10359697 | chemokine (C motif) ligand 1 | xcl1 | 0.0108 | 2.719 | up |

| **5. Colony Stimulating Factor** | | | | | |
| --- | --- | --- | --- | --- | --- |
|  |  |  |  |  |  |
| Transcripts Cluster Id | Gene description | Gene symbol | p Value | Fold Change | Regulation |
| 10456071 | colony stimulating factor 1 receptor | csf1r | 0.0219 | 2.291 | up |
| 10425066 | colony stimulating factor 2 receptor, beta, low-affinity (granulocyte-macrophage) | csf2rb | 0.0132 | 3.397 | up |
| 10430302 | colony stimulating factor 2 receptor, beta 2, low-affinity (granulocyte-macrophage) | csf2rb2 | 0.0229 | 2.276 | up |
| 10508074 | colony stimulating factor 3 receptor (granulocyte) | csf3r | 0.0233 | 3.025 | up |

| **6. CD Antigens** | | | | | |
| --- | --- | --- | --- | --- | --- |
|  |  |  |  |  |  |
| Transcripts Cluster Id | Gene description | Gene symbol | p value | FoldChange | Regulation |
| 10500406 | CD160 antigen | cd160 | 0.0160 | 1.556 | up |
| 10406928 | CD180 antigen | cd180 | 0.0007 | 5.364 | up |
| 10500677 | CD2 antigen | cd2 | 0.0027 | 3.092 | up |
| 10462390 | CD274 antigen | cd274 | 0.0216 | 16.111 | up |
| 10346783 | CD28 antigen | cd28 | 0.0179 | 2.294 | up |
| 10392845 | CD300 antigen like family member F | cd300lf | 0.0411 | 1.947 | up |
| 10562709 | CD33 antigen | cd33 | 0.0292 | 2.205 | up |
| 10584821 | CD3 antigen, delta polypeptide | cd3d | 0.0091 | 3.579 | up |
| 10593024 | CD3 antigen, epsilon polypeptide | cd3e | 0.0027 | 7.982 | up |
| 10593015 | CD3 antigen, gamma polypeptide | cd3g | 0.0433 | 3.234 | up |
| 10351658 | CD48 antigen | cd48 | 0.0163 | 4.153 | up |
| 10466040 | CD5 antigen | cd5 | 0.0019 | 2.399 | up |
| 10517165 | CD52 antigen | cd52 | 0.0043 | 7.256 | up |
| 10501063 | CD53 antigen | cd53 | 0.0192 | 4.345 | up |
| 10387536 | CD68 antigen | cd68 | 0.0418 | 2.144 | up |
| 10548333 | CD69 antigen | cd69 | 0.0301 | 2.510 | up |
| 10512470 | CD72 antigen | cd72 | 0.0085 | 2.633 | up |
| 10456005 | CD74 antigen (invariant polypeptide of major histocompatibility complex, class II antigen-associated) | cd74 | 0.0049 | 17.039 | up |
| 10439312 | CD86 antigen | cd86 | 0.0073 | 2.240 | up |
| 10538993 | CD8 antigen, alpha chain | cd8a | 0.0049 | 6.597 | up |
| 10538979 | CD8 antigen, beta chain 1 | cd8b1 | 0.0121 | 3.780 | up |
| 10557862 | integrin alpha M | Itgam | 0.0061 | 7.580 | up |

| **7. Guanylate Binding Protein (GBP)** | | | | | |
| --- | --- | --- | --- | --- | --- |
|  |  |  |  |  |  |
| Transcripts Cluster Id | Gene description | Gene symbol | p Value | Fold Change | Regulation |
| 10496555 | guanylate binding protein 1 | guanylate binding protein 5 | gbp1|gbp5 | 0.0159 | 7.116 | up |
| 10496592 | guanylate binding protein 2 | gbp2 | 0.0050 | 29.653 | up |
| 10496580 | guanylate binding protein 3 | gbp3 | 0.0117 | 16.972 | up |
| 10531987 | guanylate binding protein 4 | gbp4 | 0.0130 | 10.455 | up |
| 10496539 | guanylate binding protein 5 | gbp5 | 0.0258 | 9.826 | up |
| 10496569 | guanylate binding protein 6 | gbp6 | 0.0115 | 12.627 | up |
| 10531972 | guanylate-binding protein 8 | gbp8 | 0.0093 | 9.426 | up |
| 10531980 | guanylate-binding protein 9 | gbp9 | 0.0417 | 4.781 | up |
| 10531994 | macrophage activation 2 like | guanylate-binding protein 10 | predicted gene, EG634650 | guanylate-binding protein 8 | mpa2l|gbp10|eg634650|gbp8 | 0.0055 | 27.316 | up |

| **8. GTPases** | | | | | |
| --- | --- | --- | --- | --- | --- |
|  |  |  |  |  |  |
| Transcripts Cluster Id | Gene description | Gene symbol | p value | Fold Change | Regulation |
| 10538138 | GTPase, IMAP family member 1 | gimap1 | 0.0372 | 1.796 | up |
| 10544588 | GTPase, IMAP family member 3 | gimap3 | 0.0002 | 2.458 | up |
| 10538126 | GTPase, IMAP family member 4 | gimap4 | 0.0006 | 4.709 | up |
| 10566571 | very large inducible GTPase 1 pseudogene | GTPase, very large interferon inducible 1 | gm8979|Gm8989|Gvin1 | 0.0092 | 6.266 | up |
| 10566578 | very large inducible GTPase 1 pseudogene | GTPase, very large interferon inducible 1 | gm8979|Gvin1 | 0.0207 | 6.986 | up |
| 10566574 | GTPase, very large interferon inducible 1 | gvin1 | 0.0061 | 4.861 | up |

| **9. Interferon (IFN) activated and induced genes** | | | | | |
| --- | --- | --- | --- | --- | --- |
|  |  |  |  |  |  |
| Transcripts Cluster Id | Gene description | Gene symbol | p Value | Fold Change | Regulation |
| 10360391 | interferon activated gene 203 | ifi203 | 0.0177 | 4.280 | up |
| 10360382 | interferon activated gene 204 | myeloid cell nuclear differentiation antigen | ifi204|mnda | 0.0147 | 10.786 | up |
| 10360406 | interferon activated gene 205 | ifi205 | 0.0398 | 5.784 | up |
| 10397975 | interferon, alpha-inducible protein 27 like 1 | ifi27l1 | 0.0254 | 2.424 | up |
| 10402347 | interferon, alpha-inducible protein 27 like 2A | ifi27l2a | 0.0031 | 14.414 | up |
| 10579347 | interferon gamma inducible protein 30 | ifi30 | 0.0051 | 5.789 | up |
| 10381408 | interferon-induced protein 35 | ifi35 | 0.0433 | 4.473 | up |
| 10502791 | interferon-induced protein 44 | ifi44 | 0.0286 | 12.617 | up |
| 10375515 | interferon gamma inducible protein 47 | olfactory receptor 56 | ifi47|olfr56 | 0.0157 | 14.267 | up |
| 10462623 | interferon-induced protein with tetratricopeptide repeats 1 | ifit1 | 0.0128 | 12.479 | up |
| 10462613 | interferon-induced protein with tetratricopeptide repeats 2 | ifit2 | 0.0475 | 7.402 | up |
| 10462618 | interferon-induced protein with tetratricopeptide repeats 3 | ifit3 | 0.0152 | 9.864 | up |
| 10383756 | interferon induced transmembrane protein 2 | ifitm2 | 0.0325 | 2.235 | up |
| 10553299 | interferon induced transmembrane protein 2 | ifitm2 | 0.0150 | 2.563 | up |
| 10569014 | interferon induced transmembrane protein 2 | ifitm2 | 0.0147 | 2.534 | up |
| 10569017 | interferon induced transmembrane protein 3 | ifitm3 | 0.0094 | 3.583 | up |
| 10569020 | interferon induced transmembrane protein 6 | ifitm6 | 0.0158 | 1.702 | up |
| 10436830 | interferon (alpha and beta) receptor 2 | ifnar2 | 0.0193 | 1.681 | up |
| 10604473 | immunoglobulin superfamily, member 1 | igsf1 | 0.0162 | -1.782 | down |
| 10455961 | interferon inducible GTPase 1 | iigp1 | 0.0061 | 31.259 | up |
| 10385500 | immunity-related GTPase family M member 1 | irgm1 | 0.0128 | 17.216 | up |
| 10376326 | immunity-related GTPase family M member 2 | interferon gamma induced GTPase | irgm2|igtp | 0.0018 | 28.333 | up |
| 10554240 | interferon-stimulated protein | isg20 | 0.0309 | 5.027 | up |

| **10.Major Histocompatibility Complex** | | | | | |
| --- | --- | --- | --- | --- | --- |
|  |  |  |  |  |  |
| Transcripts Cluster Id | Gene description | Gene symbol | p Value | Fold Change | Regulation |
| 10450154 | histocompatibility 2, class II antigen A, alpha | h2-aa | 0.0070 | 19.732 | up |
| 10444291 | histocompatibility 2, class II antigen A, beta 1 | h2-ab1 | 0.0146 | 10.812 | up |
| 10450704 | histocompatibility 2, blastocyst | predicted gene 8909 | predicted gene 10499 | similar to RT1 class I histocompatibility antigen, AA alpha chain precursor | h2-bl|gm8909|Gm10499|LOC637146 | 0.0193 | 3.445 | up |
| 10444780 | histocompatibility 2, D region locus 1 | h2-d1|h2-l | 0.0048 | 8.881 | up |
| 10444229 | histocompatibility 2, class II, locus DMa | h2-dma | 0.0018 | 4.985 | up |
| 10444236 | histocompatibility 2, class II, locus Mb2 | histocompatibility 2, class II, locus Mb1 | h2-dmb2|H2-DMb1 | 0.0081 | 5.508 | up |
| 10444298 | histocompatibility 2, class II antigen E beta | h2-eb1 | 0.0066 | 11.996 | up |
| 10444814 | MHC class I like protein GS10 | histocompatibility 2, Q region locus 5 | h2-gs10|h2-q5 | 0.0088 | 11.880 | up |
| 10450075 | histocompatibility 2, K1, K region | h2-k1 | 0.0021 | 7.814 | up |
| 10444223 | histocompatibility 2, O region alpha locus | h2-oa | 0.0272 | 2.843 | up |
| 10444284 | histocompatibility 2, O region beta locus | h2-ob | 0.0274 | 1.522 | up |
| 10444788 | histocompatibility 2, Q region locus 1 | h2-q1 | 0.0083 | 1.768 | up |
| 10444841 | histocompatibility 2, Q region locus 10 | h2-q10 | 0.0061 | -2.207 | down |
| 10444824 | histocompatibility 2, Q region locus 6 | histocompatibility 2, Q region locus 6-like | h2-q6|loc68395 | 0.0174 | 4.230 | up |
| 10444830 | histocompatibility 2, Q region locus 7 | histocompatibility 2, Q region locus 6 | histocompatibility 2, Q region locus 8 | h2-q7|h2-q6|h2-q8 | 0.0158 | 8.468 | up |
| 10444821 | histocompatibility 2, Q region locus 8 | h2-q8 | 0.0002 | 5.190 | up |
| 10450682 | histocompatibility 2, T region locus 23 | RIKEN cDNA C920025E04 gene | h2-t23|c920025E04rik | 0.0086 | 14.575 | up |
| 10450675 | histocompatibility 2, T region locus 24 | h2-t24 | 0.0279 | 3.286 | up |
| 10450699 | MHC class Ib T9 | predicted gene, EG547347 | h2-t9|eg547347 | 0.0063 | 7.846 | up |

| **11. Interferon Regulatory Factors (IRF)** | | | | | |
| --- | --- | --- | --- | --- | --- |
|  |  |  |  |  |  |
| Transcripts Cluster Id | Gene description | Gene symbol | p Value | Fold Change | Regulation |
| 10376060 | interferon regulatory factor 1 | irf1 | 0.0076 | 12.207 | up |
| 10385870 | interferon regulatory factor 1 | irf1 | 0.0462 | 11.272 | up |
| 10536898 | interferon regulatory factor 5 | irf5 | 0.0002 | 2.336 | up |
| 10569102 | interferon regulatory factor 7 | irf7 | 0.0082 | 17.724 | up |
| 10576034 | interferon regulatory factor 8 | irf8 | 0.0074 | 4.448 | up |
| 10415319 | interferon regulatory factor 9 | irf9 | 0.0033 | 8.523 | up |
| 10416837 | immunoresponsive gene 1 | irg1 | 0.0109 | 4.117 | up |

| **12. Interleukins** | | | | | | | | | | |
| --- | --- | --- | --- | --- | --- | --- | --- | --- | --- | --- |
|  | |  | |  | |  |  | | |  |
| Transcripts Cluster Id | | Gene description | | Gene symbol | | p Value | Fold Change | | | Regulation |
| 10593050 | | interleukin 10 receptor, alpha | | il10ra | | 0.0001 | 4.256 | | | up |
| 10436841 | | interleukin 10 receptor, beta | | il10rb | | 0.0049 | 1.650 | | | up |
| 10572497 | | interleukin 12 receptor, beta 1 | | il12rb1 | | 0.0372 | 6.404 | | | up |
| 10599174 | | interleukin 13 receptor, alpha 1 | | il13ra1 | | 0.0491 | 1.640 | | | up |
| 10579958 | | interleukin 15 | | il15 | | 0.0388 | 1.682 | | | up |
| 10566050 | | interleukin 18 binding protein | | il18bp | | 0.0107 | 9.142 | | | up |
| 10487588 | | interleukin 1 alpha | | il1a | | 0.0388 | 2.004 | | | up |
| 10487597 | | interleukin 1 beta | | il1b | | 0.0020 | 2.302 | | | up |
| 10469816 | | interleukin 1 receptor antagonist | | il1rn | | 0.0327 | 7.328 | | | up |
| 10557342 | | interleukin 21 receptor | | il21r | | 0.0058 | 2.570 | | | up |
| 10430344 | | interleukin 2 receptor, beta chain | | il2rb | | 0.0026 | 7.361 | | | up |
| 10606016 | | interleukin 2 receptor, gamma chain | | il2rg | | 0.0032 | 7.719 | | | up |
| 10557326 | | interleukin 4 receptor, alpha | | il4ra | | 0.0094 | 2.005 | | | up |
| 10492540 | interleukin 12a | | Il12a | | 0.032 | | | 2.302 | up | |
| 10375331 | interleukin 12b | | Il12b | | 0.049 | | | 1.50969 | up | |

| **13. Lymphocyte and Leukocyte Specific Genes** | | | | | |
| --- | --- | --- | --- | --- | --- |
|  |  |  |  |  |  |
| Transcripts Cluster Id | Gene description | Gene symbol | p Value | Fold Change | Regulation |
| 10547906 | lymphocyte-activation gene 3 | lag3 | 0.0470 | 1.864 | up |
| 10559486 | leukocyte-associated Ig-like receptor 1 | lair1 | 0.0045 | 2.613 | up |
| 10559446 | leukocyte immunoglobulin-like receptor, subfamily B (with TM and ITIM domains), member 3 | lilrb3 | 0.0079 | 2.714 | up |
| 10363082 | leukocyte immunoglobulin-like receptor, subfamily B, member 4 | lilrb4 | 0.0446 | 4.438 | up |
| 10429564 | lymphocyte antigen 6 complex, locus A | ly6a | 0.0049 | 4.821 | up |
| 10429568 | lymphocyte antigen 6 complex, locus C1 | lymphocyte antigen 6 complex, locus C2 | ly6c1|ly6c2 | 0.0055 | 3.741 | up |
| 10429573 | lymphocyte antigen 6 complex, locus C2 | lymphocyte antigen 6 complex, locus C1 | ly6c2|ly6c1 | 0.0049 | 3.877 | up |
| 10424676 | lymphocyte antigen 6 complex, locus E | ly6e | 0.0009 | 2.454 | up |
| 10429560 | lymphocyte antigen 6 complex, locus I | ly6i | 0.0037 | 3.441 | up |
| 10404606 | lymphocyte antigen 86 | ly86 | 0.0142 | 4.169 | up |
| 10360158 | lymphocyte antigen 9 | ly9 | 0.0371 | 2.564 | up |

| **14. Neutrophil Specific Genes** | | | | | |
| --- | --- | --- | --- | --- | --- |
|  |  |  |  |  |  |
| Transcripts Cluster Id | Gene description | Gene symbol | p Value | Fold Change | Regulation |
| 10534202 | neutrophil cytosolic factor 1 | ncf1 | 0.0071 | 2.866 | up |
| 10425053 | neutrophil cytosolic factor 4 | ncf4 | 0.0021 | 2.318 | up |

| **15. NLR Card Domain** | | | | | |
| --- | --- | --- | --- | --- | --- |
|  |  |  |  |  |  |
| Transcripts Cluster Id | Gene description | Gene symbol | p Value | Fold Change | Regulation |
| 10574098 | NLR family, CARD domain containing 5 | nlrc5 | 0.0114 | 7.803 | up |
| 10574100 | NLR family, CARD domain containing 5 | nlrc5 | 0.0380 | 1.627 | up |
| 10574102 | NLR family, CARD domain containing 5 | nlrc5 | 0.0139 | 9.208 | up |
| 10574104 | NLR family, CARD domain containing 5 | nlrc5 | 0.0382 | 3.253 | up |
| 10574135 | NLR family, CARD domain containing 5 | nlrc5 | 0.0112 | 3.015 | up |
| 10574137 | NLR family, CARD domain containing 5 | nlrc5 | 0.0388 | 2.672 | up |
| 10574139 | NLR family, CARD domain containing 5 | nlrc5 | 0.0059 | 3.334 | up |
| 10574143 | NLR family, CARD domain containing 5 | nlrc5 | 0.0304 | 3.346 | up |
| 10574145 | NLR family, CARD domain containing 5 | nlrc5 | 0.0252 | 3.120 | up |
| 10574149 | NLR family, CARD domain containing 5 | nlrc5 | 0.0086 | 7.032 | up |
| 10574155 | NLR family, CARD domain containing 5 | nlrc5 | 0.0078 | 3.488 | up |
| 10574157 | NLR family, CARD domain containing 5 | nlrc5 | 0.0406 | 2.864 | up |
| 10574159 | NLR family, CARD domain containing 5 | nlrc5 | 0.0395 | 3.499 | up |
| 10574163 | NLR family, CARD domain containing 5 | nlrc5 | 0.0149 | 7.473 | up |
| 10388065 | NLR family, pyrin domain containing 1B | nlrp1b | 0.0132 | 1.544 | up |

| **16. T cell related genes** | | | | | |
| --- | --- | --- | --- | --- | --- |
|  |  |  |  |  |  |
| Transcripts Cluster Id | Gene description | Gene symbol | p Value | Fold Change | Regulation |
| 10441601 | T-cell activation Rho GTPase-activating protein | tagap | 0.0360 | 1.610 | up |
| 10464529 | T-cell, immune regulator 1, ATPase, H+ transporting, lysosomal V0 protein A3 | tcirg1 | 0.0149 | 1.815 | up |
| 10414817 | T-cell receptor alpha chain | RIKEN cDNA A130082M07 gene | tcra|a130082m07rik | 0.0168 | 2.080 | up |
| 10537567 | T-cell receptor beta, joining region | similar to T-cell receptor beta-2 chain C region | tcrb-J|loc665506 | 0.0215 | 2.459 | up |
| 10385518 | T-cell specific GTPase 1 | T-cell specific GTPase 2 | predicted gene 12185 | tgtp1|tgtp2|gm12185 | 0.0014 | 38.589 | up |
| 10385533 | T-cell specific GTPase 1 | T-cell specific GTPase 2 | predicted gene 12185 | tgtp1|tgtp2|gm12185 | 0.0036 | 28.285 | up |
| 10439527 | T cell immunoreceptor with Ig and ITIM domains | tigit | 0.0263 | 2.773 | up |
| 10375472 | T-cell immunoglobulin and mucin domain containing 4 | timd4 | 0.0305 | 2.300 | up |

| **17. Toll Like Receptors (TLR)** | | | | | |
| --- | --- | --- | --- | --- | --- |
|  |  |  |  |  |  |
| Transcripts Cluster Id | Gene description | Gene symbol | p Value | Fold Change | Regulation |
| 10530145 | toll-like receptor 1 | tlr1 | 0.0135 | 3.123 | up |
| 10601385 | toll-like receptor 13 | tlr13 | 0.0161 | 1.994 | up |
| 10498992 | toll-like receptor 2 | tlr2 | 0.0452 | 2.482 | up |
| 10505517 | toll-like receptor 4 | tlr4 | 0.0219 | 2.353 | up |
| 10607870 | toll-like receptor 7 | tlr7 | 0.0309 | 2.127 | up |
| 10588479 | toll-like receptor 9 | tlr9 | 0.0051 | 3.953 | up |

| **18. Tumor Necrosis Factor (TNF)** | | | | | |
| --- | --- | --- | --- | --- | --- |
|  |  |  |  |  |  |
| Transcripts Cluster Id | Gene description | Gene symbol | p | Fold | Regulation |
| 10450501 | tumor necrosis factor | tnf | 0.0182 | 2.527 | up |
| 10368144 | tumor necrosis factor, alpha-induced protein 3 | tnfaip3 | 0.0271 | 1.622 | up |
| 10500100 | tumor necrosis factor, alpha-induced protein 8-like 2 | tnfaip8l2 | 0.0035 | 2.258 | up |
| 10519060 | tumor necrosis factor receptor superfamily, member 14 (herpesvirus entry mediator) | tnfrsf14 | 0.0357 | 1.504 | up |
| 10511290 | tumor necrosis factor receptor superfamily, member 18 | tnfrsf18 | 0.0356 | 2.586 | up |
| 10541895 | tumor necrosis factor receptor superfamily, member 1a | tnfrsf1a | 0.0469 | 2.042 | up |
| 10518300 | tumor necrosis factor receptor superfamily, member 1b | tnfrsf1b | 0.0048 | 2.664 | up |
| 10576951 | tumor necrosis factor (ligand) superfamily, member 13b | predicted gene 10703 | tnfsf13b|gm10703 | 0.0086 | 1.521 | up |
